# Proton-Pump Inhibitors and Serum Concentrations of Uremic Toxins in Patients with Chronic Kidney Disease

**DOI:** 10.3390/toxins15040276

**Published:** 2023-04-07

**Authors:** Carolla El Chamieh, Islam Amine Larabi, Solène M. Laville, Christian Jacquelinet, Christian Combe, Denis Fouque, Maurice Laville, Luc Frimat, Roberto Pecoits-Filho, Céline Lange, Bénédicte Stengel, Natalia Alencar De Pinho, Jean-Claude Alvarez, Ziad A. Massy, Sophie Liabeuf

**Affiliations:** 1 Centre for Research in Epidemiology and Population Health (CESP), INSERM UMRS 1018, Université Paris-Saclay, Université Versailles Saint Quentin, 94807 Villejuif, France; carolla.el-chamieh@inserm.fr (C.E.C.); christian.jacquelinet@biomedecine.fr (C.J.); celine.lange@biomedecine.fr (C.L.); benedicte.stengel@inserm.fr (B.S.); natalia.alencar-de-pinho@inserm.fr (N.A.D.P.); ziad.massy@aphp.fr (Z.A.M.); 2 Department of Pharmacology and Toxicology, Raymond Poincaré Hospital, AP-HP, 92380 Garches, France; islamamine.larabi@aphp.fr (I.A.L.); jean-claude.alvarez@aphp.fr (J.-C.A.); 3 UVSQ, Université Paris-Saclay, Inserm U1018, CESP, Équipe MOODS, MasSpecLab, 78180 Montigny-le-Bretonneux, France; 4 Pharmacoepidemiology Unit, Department of Clinical Pharmacology, Amiens-Picardie University Medical Center, 80054 Amiens, France; laville.solene@chu-amiens.fr; 5 MP3CV Laboratory, Jules Verne University of Picardie, F-80054 Amiens, France; 6 Biomedecine Agency, 93210 Saint Denis La Plaine, France; 7 Service de Néphrologie Transplantation Dialyse Aphérèse, Centre Hospitalier Universitaire de Bordeaux, 33076 Bordeaux, France; christian.combe@chu-bordeaux.fr; 8 INSERM, U1026, Univ. Bordeaux, 33076 Bordeaux, France; 9 Nephrology Department, Centre Hospitalier Lyon Sud, Université de Lyon, Carmen, 69495 Pierre-Bénite, France; denis.fouque@univ-lyon1.fr; 10 Université de Lyon, CarMeN INSERM 1060, 69008 Lyon, France; maurice.laville@chu-lyon.fr; 11 Nephrology Department, CHRU de Nancy, 54000 Vandoeuvre-lès-Nancy, France; l.frimat@chu-nancy.fr; 12 Lorraine University, APEMAC, 54000 Vandoeuvre-lès-Nancy, France; 13 Arbor Research Collaborative for Health, Ann Arbor, MI 48108, USA; roberto.pecoits@arborresearch.org; 14 School of Medicine, Pontificia Universidade Catolica do Parana, Curitiba 80215-901, Brazil; 15 Department of Nephrology, Ambroise Paré University Hospital, APHP, 92104 Boulogne-Billancourt, France

**Keywords:** uremic toxins, indoxyl sulfate, proton-pump inhibitor, chronic kidney disease

## Abstract

Use of proton-pump inhibitors (PPIs) is common in patients with chronic kidney disease (CKD). PPIs and many uremic toxins (UTs) are eliminated by the kidney’s tubular organic anion transporter system. In a cross-sectional study, we sought to evaluate the association between PPI prescription and serum concentrations of various UTs. We studied a randomly selected sub-group of participants in the CKD-REIN cohort (adult patients with a confirmed diagnosis of CKD and estimated glomerular filtration rate (eGFR) < 60 mL/min/1.73 m^2^) with available frozen samples collected at baseline. PPI prescription was recorded at baseline. Serum concentrations of 10 UTs were measured using a validated liquid chromatography tandem mass spectrometry technique. Multiple linear regression was performed, with the log UT concentration as the dependent variable. Of the 680 included patients (median age: 68 years; median eGFR: 32 mL/min/1.73 m^2^), 31% had PPI prescriptions at baseline. Patients using PPIs had higher levels of certain UTs in comparison to other patients, including total and free indoxyl sulfate (IS), total and free p-cresylsulfate, total and free p-cresylglucuronide (PCG), phenylacetylglutamine (PAG), free kynurenine, and free hippuric acid. After adjustment for baseline co-morbidities, number of co-prescribed drugs, and laboratory data, including eGFR, associations between PPI prescription and elevated serum concentrations of free and total IS, free and total PCG, and PAG remained significant. Our results indicate that PPI prescription is independently associated with serum UT retention. These findings are interesting to better understand the factors that may modulate serum UT concentration in CKD patients, however, they will need to be confirmed by longitudinal studies.

## 1. Introduction

Chronic kidney disease (CKD) is a global health burden affecting between 8 and 16% of the adult population worldwide [1,2,3,4]. It is a progressive, irreversible condition that is responsible for a non-negligible rate of complications, affecting the quality of life and increasing hospitalization rates. Cardiovascular and overall mortality risks are increased with the progression of CKD [3].

This public health issue is characterized by the accumulation of uremic toxins (UTs) as the level of kidney function decreases. In fact, UTs constitute one of the non-traditional risk factors associated with the progression of CKD and an elevated incidence of cardiovascular (CV) morbidity and mortality, neurotoxicity, and/or bone disease [2,5]. Hence, there is a fundamental need to identify factors that can impact UT concentrations.

Many UTs and drugs administered to CKD patients are organic anions, which are physiologically eliminated by the organic anion transporter (OAT) system [6,7]. OATs (mainly expressed in the kidney, liver, and brain) are known to be involved in the transportation of a number of commonly prescribed drugs, such as proton-pump inhibitors (PPIs), diuretics, and antibiotics [8,9]. Moreover, the results of recent in vitro and in vivo studies have demonstrated that over 35 UTs (especially the protein-bound toxins, such as indoxyl sulfate (IS) and p-cresyl sulfate (PCS)) are substrates for the main renal toxin and drug transporters OAT1 (also known as SLC22A6) and OAT3 (also known as SLC22A8) expressed in the proximal tubule in the kidney [10,11]. Therefore, potential UT–drug competition might explain changes in the concentrations of certain UTs and might accentuate toxic cascade effects.

A few studies have shown that the inhibition of OAT1/OAT3 transporters (whether renal or neuronal) induces the accumulation of UTs [7,8,12]. For example, an in vivo study in rats demonstrated that the administration of ciprofloxacin decreased the renal clearance of IS [13].

Patients with CKD usually suffer from multiple co-morbidities. Thus, polypharmacy is highly prevalent in this population and frequently involves PPIs [14]. The latter drugs are well known to be OAT1/OAT3 inhibitors; one can therefore reasonably hypothesize that PPIs and UTs interact. The adverse outcomes associated with PPI use in CKD patients have been investigated, with associations between PPI prescription and acute kidney injury (AKI), progression to end-stage kidney disease, and mortality [14].

To the best of our knowledge, the association between PPI prescription and serum UT concentrations has not previously been investigated in patients with non-dialysis-dependent (NDD) CKD. Our starting hypothesis was that serum UT concentrations are higher in patients using PPIs than in those who do not, and that this association is independent of other determinants of UT level including estimated glomerular filtration rate (eGFR). The main objective of the present cross-sectional study was to evaluate a putative, independent association between PPI prescription and baseline serum concentrations of various UTs in a sub-cohort of patients with NDD CKD, after adjustment for the eGFR and other confounding factors.

## 2. Results

### 2.1. Baseline Patients’ Characteristics

A total of 680 patients were analyzed in this study, with overall patients’ characteristics described in Table 1. The median [interquartile range (IQR)] age of the participants was 68 [61–77] years, 31% were women, and the median body mass index (BMI) was 28 [25.2–31.4] kg/m^2^. A large proportion of the participants had diabetes (40%). Almost all the participants had hypertension (96.6%). Of the participants, 49% were former smokers and 21% presented a history of AKI. Thirty-one percent of patients had a PPI prescription at inclusion. The median eGFR was 32 [23–41] mL/min/1.73 m^2^. PPI users differed significantly from non-users with regard to baseline characteristics. Patients with a PPI prescription were significantly older, more likely to be female, had higher serum high-sensitivity C-reactive protein (CRP) levels, a greater prevalence of co-morbidities, a greater median total number of medications, and lower serum levels of eGFR, albumin, and hemoglobin (*p* < 0.05).

The distribution of log-normalized UT serum levels by CKD stage is shown in Figure S1 in the Supplementary Material. Patients with more advanced CKD had significantly higher UT concentrations, except for the free 3-carboxy-4-ethyl-5-propyl-2-furanpropanoic acid (CMPF) fraction.

The three most prescribed PPIs in our study are esomeprazole, lansoprazole, and omeprazole (Table 2).

### 2.2. Crude Analysis: Associations between Uremic Toxins and Proton-Pump Inhibitors

Table 3 summarizes the distribution of each UT concentration in the study population and the comparisons of their respective levels according to PPI prescription. Both free and total fractions of UTs are given for protein-bound UTs.

The median serum levels of free and total IS, free kynurenine, free and total PCS, free and total p-cresyl glucuronide (PCG), phenylacetylglutamine (PAG), and free hippuric acid (HA) were significantly higher for PPI users than for the other patients.

We did not observe significant intergroup differences for free and total indole-3-acetic acid (IAA), total kynurenine, total kynurenic acid (KA), total HA, free and total CMPF, trimethylamine N-oxide (TMAO), or urea. Hence, possible associations between these variables and PPIs were not assessed further by adjustment for confounding factors in multiple regression analysis.

### 2.3. Adjusted Analyses: Associations between Uremic Toxins and Proton-Pump Inhibitors

The multiple linear regression analysis (showed in Table 4) confirmed the association between PPIs and elevated serum concentrations of free and total IS, free and total PCG, and PAG, even after adjustment for age, sex, the total number of co-prescribed medications, a history of AKI, serum CRP and albumin levels, diabetes, BMI, smoking status, urine albumin-to-creatinine ratio or protein-to-creatinine ratio (ACR), and eGFR.

However, after adjustment for the same confounding factors, free kynurenine, free and total PCS, and free HA were no longer significantly associated with PPI use (Table 4).

## 3. Discussion

In this cross-sectional study, we sought to evaluate the association between PPI use (well-known OAT1/OAT3 inhibitors) and baseline concentrations of serum UTs in a randomly selected sub-group of the nationwide CKD-REIN cohort of patients with moderate-to-advanced CKD under nephrology care. Our study is the first to show that (i) serum levels of PAG, free and total IS, and free and total PCG are significantly higher in patients taking PPIs than in those who do not and (ii) this association remains statistically significant after adjustment for eGFR, age, sex, history of AKI, serum CRP and albumin levels, diabetes, BMI, smoking status, ACR, and the total number of co-prescribed medications.

It is well known that UTs are associated with CKD progression and several CKD-related complications (e.g., neurological complications and bone diseases) and a rise in mortality rates. Furthermore, a growing body of evidence shows that UTs (mainly IS) have a major role in the development of atheromatous and/or non-atheromatous CV diseases—the leading cause of death in patients with CKD [2].

A better understanding of the factors responsible for modulating serum UT concentrations (independently of the effect of renal dysfunction) is essential for limiting the harmful effects of UTs, improving life expectancy among patients with CKD, and facilitating the development of novel therapeutic strategies.

PPIs are among the world’s most frequently prescribed drugs—especially in patients with CKD, where the prevalence varies from 30% to 45% [14,15]. The use of PPIs by patients with CKD is commonly associated with polypharmacy and/or with concomitant antithrombotic drugs [14]. However, PPIs may be overprescribed and are often inappropriately used [16]. Although PPIs are considered to be effective and medically important for many patients, long-term prescription of these drugs needs to be re-evaluated with a view to avoiding both non-serious and serious long-term adverse drug reactions [17]. Several studies have described the direct toxicity of PPIs. Long-term PPI intake is associated with osteoporosis, hypomagnesemia, AKI, CKD progression, drug interactions, and mortality [18,19]. Our group has recently found that new PPI intake was associated with an increased risk of AKI and progression to kidney failure [14]. Xie et al. also showed that PPI intake is significantly associated with elevated mortality [20].

Notably, affinity to OATs varies between UTs [10] and according to the type and/or dose of PPI [21,22,23,24,25,26] ( Supplementary Tables S2 and S3). The three most prescribed PPIs in our study seem to have high affinity for OAT3 and two of them strongly high affinity for OAT1, concluding on their high potential effect in modulating serum concentrations of UTs that are also eliminated by the transport system OAT1/3. In the present analysis, we reported that PPIs are associated with serum UT concentrations, the potential interaction between PPIs and UTs had been poorly investigated in the literature. A recent study of kidney transplant recipients showed that the use of at least one OAT1/OAT3 inhibitor (such as a PPI) was associated with the accumulation of certain UTs that are substrates for these transporters [12]. Likewise, the results of a few animal studies show that some OAT1/OAT3-inhibiting drugs decrease the excretion of UTs (notably IS) [13,27].

Among the UTs known to be transported by OAT1/OAT3 and assessed in our study, only serum levels of IS were significantly elevated in patients using PPIs—even after considering confounding factors such as the eGFR. No previous preclinical or clinical studies have shown this specific association. However, the few in vivo studies to have evaluated drug–UT interactions for OATs concluded that IS clearance is decreased by the administration of certain drugs transported by OAT1 or OAT3. The drugs that interact with OAT1 and OAT3 include β-lactam antibiotics, diuretics, uricosuric agents, and non-steroidal anti-inflammatory drugs [28]. A recent study of nephrectomized rats by Luo et al. demonstrated that the administration of ciprofloxacin was associated with less renal IS excretion [13]. In Granados et al.’s study of a cohort of 20 probenecid-treated subjects, high levels of tryptophan-derived metabolites (including IS, IAA, and kynurenine) were observed [29]. Probenecid is an uricosuric drug used in the treatment of gout and also used in preclinical trials as a positive control for OAT1/OAT3 inhibition [28]. Another animal study by Yu et al. concluded that the nonsteroidal anti-inflammatory drugs diclofenac and ketoprofen inhibited the clearance of IS by inhibiting OAT1/OAT3-mediated uptake of IS [27]. However, in contrast to PPIs, the latter drugs are rarely prescribed to patients with CKD [30]. As mentioned above, PPIs are also considered to be OAT inhibitors; hence, in mechanistic terms, one can reasonably hypothesize that PPIs and IS interact through OATs and thus that PPI use increases serum IS levels.

However, we found no significant association between other UTs transported by OAT1/OAT3 included in our study (IAA, kynurenine, KA, PCS, HA, and CMPF) and PPI prescription. These results might be influenced by the dose of each PPI, a factor that was not included in our analysis and that could interact differently with each UT serum concentration. PPIs have a dose-dependent effect and a concentration-dependent manner to block OATs [21]. Independently, each PPI and UT has different affinity to OATs and different binding regions. The specific UT affinity to OATs might also be modulated by other OAT inhibitor drugs, such as diuretics that may have different capacity to interact with OATs [12].

A study has shown that probenecid significantly reduces the uptake of PCS by renal cells [31]. PCS accumulation was also confirmed in a recent clinical study by André et al. [12], who concluded that the prescription of at least one OAT1/OAT3 inhibitor was independently associated with the serum accumulation of PCS after adjusting for eGFR, age, the plasma albumin level, and time since transplantation. Our study revealed a significant crude association between PPI prescription and elevated serum free PCS concentrations, but this association was no longer significant after adjustment. PCS retention caused by OAT-excreted medications other than PPIs might explain the disparity between André et al.’s [12] findings and our negative result.

Furthermore, we found that PPI prescription was significantly associated with elevated serum concentrations of PAG and both free and total PCG, after adjustment for confounding factors. For instance, it has not been shown that PCG and PAG are transported by OATs. Nevertheless, several studies have demonstrated that PPIs can modulate the composition and metabolism of the gut microbiota [32]. These changes might modulate concentrations of UTs formed in the digestive tract, such as PCG, PAG, and IS [2,33]. However, the enzymes involved in the gut microbiota metabolism have not yet been investigated, and preclinical studies are warranted to explain and validate this possible mechanism. Regarding other gut-derived UTs that are not OAT substrates, no association was found with PPI, assuming that other confounding factors could influence serum UT levels such as the dietary index. Plasma levels of dietary and gut-derived UTs might have been modulated by the content of dietary intake (protein and fiber) [34].

The present study had some strengths. Although we did not assess all the participants in the CKD-REIN cohort (given the cost and time-consuming nature of the UT assays), this representative number was still large enough to enable the inclusion of a sufficient number of confounders in the multiple analysis and to have enough statistical power. More importantly, all UTs were quantified in the same laboratory, resulting in centralized UT assays that were measured using the validated robust ultrahigh-performance liquid chromatography tandem mass spectrometry (LC-MS/MS) technique.

While analyzing our data, it is important to keep in mind a few limitations. Firstly, our cross-sectional design had some drawbacks. Given the observational type of our study, we cannot conclude on any causal relationship between the observed associations. Consequently, longitudinal studies are needed to confirm our results, and preclinical studies must be conducted to explain the pathogenic mechanisms. Secondly, our results might have been influenced by residual confounding, given that unknown risk factors might not have been included in our analyses. Nevertheless, when evaluating the independent association of PPI with serum UT levels, we adjusted for many major parameters known to influence UTs, PPIs, and gut microbiota including renal function. Lastly, our definition of PPI exposure was based on prescriptions, without considering the effects of PPI dose [21] or duration of therapy prior to the baseline interview.

In conclusion, this study is the first to indicate that the PPIs commonly prescribed to CKD patients are linked to elevated serum UT concentrations (namely IS, PCG, and PAG, in the present work). Given the association between UTs and CKD-related morbidity and mortality, our findings are interesting to better understand the potential factors that may modulate serum UT concentration in CKD patients, however, they will need to be confirmed by longitudinal studies.

## 4. Materials and Methods

### 4.1. Study Design and Participants

The Chronic Kidney Disease–Renal Epidemiology and Information Network (CKD-REIN) is a large prospective cohort in 40 representative nephrology outpatient clinics in France. In brief, the eligibility criteria were age 18 or over, a confirmed diagnosis of moderate or advanced CKD (eGFR < 60 mL/min/1.73 m^2^), and no prior maintenance dialysis or kidney transplantation. Between July 2013 and April 2016, 3033 adult patients gave their informed consent to participate in the cohort and were included for five years of annual follow-up. Details of the study protocol have been published elsewhere [35].

The present study is based on a sub-cohort of 30% of the CKD-REIN participants selected at random, giving a total of 680 participants whose characteristics are comparable to those of the overall cohort (except for a slight difference for sex) (Supplementary Table S1).

Data were collected by trained clinical research associates from medical records or patient interviews at baseline and then annually.

### 4.2. Medication Exposure

Information on medication use (including PPIs) was obtained from drug prescriptions during the baseline interview. The PPIs included omeprazole, esomeprazole, pantoprazole, lansoprazole, and rabeprazole. Dates of PPI prescription are concomitant with the dates of UT serum sampling; all patients considered as PPI users have a PPI prescription at the date of serum sampling.

### 4.3. Outcome: Quantification of UTs

The study’s primary outcome was the serum levels of UT at baseline. Indeed, upon enrollment of the patients, serum samples were collected, immediately stored at 4 °C, and aliquoted within 6 h without additional processing. All CKD-REIN samples were stored frozen (−80 °C) at the Biobanque de Picardie (BRIF number: BB-0033-00017) and shipped frozen to Paris for analysis. Both teams were blind to outcome and patients’ characteristics. UT fractions were assayed in serum using a previously validated LC-MS/MS technique, as described previously [36]. In the CKD-REIN cohort, serum levels of the following 10 UTs were assessed: IS, IAA, kynurenine, KA, PCS, PCG, HA, CMPF, TMAO, and PAG. Among these UTs, TMAO and PAG are small, water-soluble molecules, while the others are protein-bound molecules. Therefore, serum concentrations were reported as free and total only for the protein-bound UTs.

To determine total UT (free and protein-bound) concentrations, 50 µL of serum samples was first precipitated with 340 µL of methanol in the presence of 25 µL of isotolabeled internal standards. After centrifugation for 10 min at 9000× *g*, the supernatant was evaporated under nitrogen stream and then reconstituted in 80 µL of water.

Free UT concentrations were determined by ultrafiltration; 150 µL of serum was introduced into an ultracentrifugal filter of 30 kDa porosity and then centrifuged at 13,300× *g* for 20 min. Given that UTs are mainly bound to albumin (65 kDa, which does not pass through the filter), the residual filtrate contained only the free UT fraction. Free KA was below the limit of detection (<0.01 mg/L) in most of the patients and so was not included in our analysis.

### 4.4. Covariates

We collected the patients’ characteristics (age, sex, and smoking status) and detailed medical history, including any history of diabetes, hypertension, dyslipidemia, CV disease, and AKI. Patients were classified with a history of CV disease if they had a history of coronary heart disease, stroke, peripheral vascular disease, heart failure, dysrhythmia, or valvular disease. They were classified as having diabetes if the condition was mentioned in their medical records, if they had an HbA1c level ≥ 7%, or if they used glucose-lowering medication. Likewise, patients were classified as having hypertension if this was mentioned in their medical records, if they had a measured systolic blood pressure ≥ 140 mm Hg or a diastolic blood pressure ≥ 90 mm Hg or if they used antihypertensive medication, and they were classified as having dyslipidemia if it was stated in their medical records or if they used lipid-lowering medication. Nephrologists or outpatient nurses measured the patients’ blood pressure, weight, and height and calculated the BMI. All patients were prescribed standard blood and urine tests (as recommended by the French health authorities for routine CKD care), which were performed at their usual laboratory at study entry, or at a centralized laboratory for the measurement of CRP and creatinine. We recorded data on the serum hemoglobin, CRP, urea, creatinine, and albumin levels and the ACR. The eGFR was calculated from serum creatinine using the Chronic Kidney Disease–Epidemiology Collaboration (CKD-EPI) equation [37], and the ACR was classified according to the Kidney Disease Improving Global Outcomes (KDIGO) guidelines [38].

### 4.5. Statistical Analysis

Continuous variables are quoted as the median [IQR] or the mean (standard deviation) as appropriate, and categorical variables are quoted as the percentage. The Wilcoxon rank sum test or Student’s *t*-test was used (as appropriate) to compare continuous variables in two groups, the chi-squared test was used to compare two categorical variables. Given that the UT concentration values were not normally distributed, they were log-transformed prior to tests that assume a Gaussian distribution. The Kruskal–Wallis test was used to compare the levels of each UT in the various CKD stages, followed by a post hoc analysis using Dunn’s test (Supplementary Figure S1).

For each studied UT, a linear regression was performed to assess the crude association between PPI prescription and UT levels. Each UT–PPI association found to be statistically significant in the crude analysis was further assessed in a multiple analysis approach. A multiple linear regression was performed for each UT with the log UT concentration as the dependent variable. Clinically relevant variables preselected following a review of the literature and those with *p*  <  0.2 in the univariate analysis were included in the multiple analysis model: age at inclusion, sex, serum CRP level, history of AKI, smoking status, albuminemia, diabetes, BMI, total number of co-prescribed medications, ACR, and eGFR at inclusion. The assumptions required by the linear regression analysis were checked and met in all cases.

Patients whose urea concentration values were considered to be outliers (serum urea <2.5 mM or >100 mM) were considered as missing data.

Assuming that data were missing at random, we performed multiple imputation with chained equations (MICE) [39], implemented with the MICE package in R software (version 4.1.2, R Foundation for Statistical Computing, Vienna, Austria) [40]. Given the proportion of missing data, 32 complete datasets were imputed. The imputation model included all the variables present in the models (UTs, PPI, and each model’s covariates). Linear regression models were generated for each dataset, and pooled regression coefficients were obtained according to Rubin’s rules. The threshold for statistical significance was *p* < 0.05. All statistical analyses were performed with R software.

## Figures and Tables

**Table 1:** Baseline characteristics of the study population.

Characteristics	Total (*n* = 680) ^a^	PPI Use (*n* = 211) ^a^	No PPI Use (*n* = 469) ^a^	*p*-Value ^b^	Imputed Data
Age at baseline (years)	68 [61–77]	71 [65–78]	67 [59–75]	**<0.001**	0%
Women	31%	37%	28%	**0.02**	0%
eGFR at baseline (mL/min/1.73 m^2^)	32 [23–41]	30 [22–39]	32 [24–42]	**0.02**	0%
Albuminuria categories				0.38	8%
A1 (Normal to mildly increased)	30%	27%	31%		
A2 (Moderately increased)	30%	33.5%	28%		
A3 (Severely increased)	40%	39.5%	41%		
History of acute kidney injury	21%	28%	17.5%	**0.002**	7.5%
Smoking status					
Never smoker	38%	39.5%	37.5%	0.69	0.7%
Current smoker	13%	13.5%	12%		
Former smoker	49%	47%	50.50%		
Hypertension	96.6%	98%	96%	0.16	0.3%
Diabetes	40%	44%	38.5%	0.16	0.3%
Dyslipidemia	73%	79.4%	69.6%	**0.008**	0.4%
History of cardiovascular disease	52%	63.5%	47.3%	**<0.001**	0.6%
Serum albumin (g/L)	40.5 [37.8–43]	40 [37.5–42]	41 [38–43]	**0.03**	14%
Hemoglobin (g/dl)	13.1 (1.64)	12.8 (1.7)	13.2 (1.5)	**0.002**	0.6%
High-sensitivity C-reactive protein (mg/L)	2 [1–6]	4 [2–8]	2 [1–5]	**<0.001**	0%
Body mass index (kg/m^2^)	28 [25.2–31.4]	28.5 [25.1–32.4]	27.8 [25.2–31]	0.38	1.1%
Total number of medications	8 [5–10]	10 [8–12]	7 [4–9]	**<0.001**	0.29%

^a^ Median [interquartile range] or mean (standard deviation); %; ^b^ Wilcoxon rank sum test or *t*-test; Pearson’s chi-squared test.

**Table 2:** Description of types of PPI prescription.

Types of PPI	PPI Prescription (*n* = 211) ^a^
**Omeprazole**	41 (19.5%)
**Esomeprazole**	100 (47.5%)
**Pantoprazole**	11 (5%)
**Lansoprazole**	48 (23%)
**Rabeprazole**	11 (5%)

^a^ *n* (%).

**Table 3:** Median serum uremic toxin concentrations according to PPI use at baseline.

Uremic Toxin	Total (*n* = 680) ^a^	PPI Use (*n* = 211) ^a^	No PPI Use (*n* = 469) ^a^	*p*-Value ^b^	Imputed Data
Indoxyl sulfate (mg/L)					0%
Free	0.06 [0.03–0.1]	0.08 [0.04–0.14]	0.05 [0.03–0.1]	**<0.001**	
Total	4.7 [2.7–8.0]	5.7 [3.3–10.60]	4.3 [2.4–7.20]	**<0.001**	
Indole-3-acetic acid (mg/L)					0%
Free	0.034 [0.023–0.053]	0.035 [0.024–0.056]	0.034 [0.023–0.05]	0.31	
Total	0.59 [0.38–0.88]	0.57 [0.37–0.80]	0.6 [0.4–0.90]	0.6	
Kynurenine (mg/L)					0%
Free	0.15 [0.10–0.20]	0.2 [0.1–0.22]	0.15 [0.1–0.2]	**0.04**	
Total	1.40 [1.07–1.86]	1.44 [1.04–1.90]	1.38 [1.08–1.80]	0.97	
Kynurenic acid (mg/L)					0%
Free	<LOD	NA	NA		
Total	0.029 [0.018–0.043]	0.03 [0.02–0.04]	0.03 [0.02–0.04]	0.54	
P-cresyl sulfate (mg/L)					0%
Free	0.22 [0.11–0.47]	0.31 [0.15–0.57]	0.20 [0.09–0.41]	**<0.001**	
Total	16 [8–27]	18 [11–30]	15 [8–25]	**0.003**	
P-cresyl glucuronide (mg/L)					0%
Free	0.06 [0.02–0.13]	0.09 [0.04–0.17]	0.05 [0.02–0.11]	**<0.001**	
Total	0.08 [0.03–0.19]	0.13 [0.05–0.24]	0.06 [0.03–0.16]	**<0.001**	
Hippuric acid (mg/L)					0%
Free	0.91 [0.44–1.91]	1.05 [0.50–2.07]	0.86 [0.42–1.78]	**0.025**	
Total	3.7 [1.9–7.8]	4.2 [2.1–8.2]	3.5 [1.8–7.3]	0.17	
3-carboxy-4-methyl-5-propyl-2-furanpropanoic acid (mg/L)					0%
Free	0.011 [0.006–0.017]	0.010 [0.005–0.016]	0.011 [0.006–0.017]	0.11	
Total	2.9 [1.4–5.5]	2.6 [1.2–5.2]	3.0 [1.6–5.7]	0.06	
Phenylacetylglutamine (mg/L)	2.55 [1.31–4.25]	3.21 [1.87–5.44]	2.22 [1.17–3.74]	**<0.001**	0%
Trimethylamine N-oxide (mg/L)	1.48 [0.87–2.56]	1.49 [0.86–2.54]	1.48 [0.87–2.56]	0.9	0%
Urea (mmol/L)	12.6 [9.5–17.5]	12.8 [9.8–17.3]	12.4 [9.5–17.5]	0.57	6.10%

^a^ Median [interquartile range]. ^b^ Wilcoxon rank sum test. Statistically significant *p*-values are given in bold type. LOD: limit of detection (0.01 mg/L). NA: not applicable.

**Table 4:** Crude and adjusted coefficients of log uremic toxin concentrations associated with proton-pump inhibitors.

Log Uremic Toxin	Crude Analysis		Adjusted Analysis	
	Beta [95% CI]	*p*-Value	Beta [95% CI]	*p*-Value ^a^
Free indoxyl sulfate	0.40 [0.24, 0.57]	<0.001	0.19 [0.02, 0.35]	**0.02**
Total indoxyl sulfate	0.37 [0.22, 0.51]	<0.001	0.24 [0.1, 0.3]	**0.001**
Free kynurenine	0.10 [0.02, 0.18]	0.02	0.01 [−0.1, 0.07]	0.7
Free p-cresyl sulfate	0.38 [0.19, 0.58]	<0.001	0.05 [−0.1, 0.2]	0.6
Total p-cresyl sulfate	0.23 [0.06, 0.39]	0.003	0.002 [−0.17, 0.17]	0.9
Free p-cresyl glucuronide	0.51 [0.33, 0.70]	<0.001	0.24 [0.05, 0.4]	**0.01**
Total p-cresyl glucuronide	0.52 [0.33, 0.72]	<0.001	0.23 [0.02, 0.4]	**0.02**
Free hippuric acid	0.20 [0.03, 0.37]	0.02	0.13 [−0.04, 0.3]	0.1
Phenylacetylglutamine	0.36 [0.22, 0.50]	<0.001	0.14 [0.09, 0.2]	**0.03**

^a^ Each model was adjusted for: age, sex, total number of co-prescribed medications, history of AKI, serum CRP, serum albumin, diabetes, BMI, smoking status, ACR categories, and eGFR (in mL/min per 1.73 m^2^). Statistically significant *p*-values are given in bold type.

## Data Availability

The data presented in this study are available on request from the corresponding author. The data are not publicly available due to confidential reasons of the national CKD-REIN study.

## Supplementary Materials

The following supporting information can be downloaded at: https://www.mdpi.com/article/10.3390/toxins15040276/s1. STROBE statement; Table S1: Characteristics comparison between included and non-included patients in the sub-cohort; Table S2: Different types of PPI and their affinity to OAT1/3; Table S3: Affinity of uremic toxins to OAT1/3; Figure S1: Distribution of log-normalized uremic toxins according to the stages of chronic kidney disease; Figure S2: Distribution of log-normalized uremic toxins according to proton-pump inhibitor use; list of biological resources centers.
