# Supplementary material for: Proton-Pump Inhibitors and Serum Concentrations of Uremic Toxins in Patients with Chronic Kidney Disease
Source: Toxins (Basel). 2023 Apr 7;15(4):276. doi: 10.3390/toxins15040276 (PMC10143607; doi:10.3390/toxins15040276)
Supplement: Supplementary file 1 [file toxins-15-00276-s001.zip › toxins-2303763-supplementary.pdf]

Table S1. Characteristics comparison between included and non-included patients in the sub-cohort

Characteristics	Overall (N = 3033) [a]	Included in the sub-cohort (n=680) [a]	Not included in the sub-cohort (n=2353) [a]	*P*-value [b]
**Age at baseline (years)**	69 [60-76]	68 [61-77]	69 [60-76]	0.77
**Men**	65.4%	69%	64.3%	0.02
**eGFR at baseline (ml/min/1.73 m^2)**	32 [23-41]	32 [23-41]	32 [23-41]	0.89
**Albuminuria categories**				0.39
A1 (Normal to mildly increased)	27.8%	30%	27.2%	
A2 (Moderately increased)	31.2%	30%	31.6%	
A3 (Severely increased)	40.9%	40%	41.1%	
NA	275	58	217	
**History of acute kidney injury**	23.6%	21%	24.4%	0.08
NA	244	51	193	
**Smoking status**				0.17
Never-smoker	41.3%	38%	42%	
Current smoker	11.9%	13%	12%	
Former smoker	46.8%	49%	46%	
NA	23	5	18	
**Hypertension**	96.3%	96.6%	96.2%	0.64
NA	7	2	5	
**Diabetes**	43.2%	40%	44%	0.08
NA	7	2	5	
**Dyslipidemia**	73.6%	73%	73.9%	0.46
NA	14	3	11	
**History of cardiovascular disease**	53.3%	52%	53.6%	0.52
NA	42	4	38	
**Serum albumin (g/l)**	40.3 [38.0-43.0]	40.5 [37.8-43]	40.2 [38.0-43.0]	0.91
NA	489	94	395	
**Hemoglobin (g/dl)**	13 (1.65)	13.1 (1.64)	12.9 (1.65)	0.07
NA	23	4	19	
**High-sensitivity C-reactive protein (mg/l)**	2 [1-6]	2 [1-6]	3 (1, 6)	0.92
NA	356	0	356	
**Body mass index (kg/m^2)**	27.8 [24.6-31.8]	28 [25.2-31.4]	27.8 [24.5-31.9]	0.56
NA	65	8	57	
**Proton pump inhibitor**	32.8%	31%	33.3%	0.25
NA	7	0	7	

**Total number of medications**	8 [5-10]	8 [5-10]	8 (5, 10)	0.36
NA	9	2	7	

[a] Median [interquartile range] or mean (standard deviation); %
[b] Wilcoxon rank sum test; Pearson's Chi-squared test

Table S2. Different types of PPI and their affinity to OAT1/3

Types of PPI	Plasma Protein Binding [22]	Affinity to OAT1	Affinity to OAT3
Omeprazole	95-96%	Strongly high affinity [21, 23]	High affinity [21-24]
Esomeprazole	97%	Limited data [23]	High affinity [22-24]
Pantoprazole	92%	Moderate affinity [21]	High affinity [21,22,24]
Lansoprazole	97%	Strongly high affinity [21]	High affinity [21-26]
Rabeprazole	97%	Limited data [23]	High affinity [22,23]

[a] n (%)

Table S3. Affinity of uremic toxins to OAT1/3

Uremic toxin	Affinity to OAT1/3 [10]	Gut-derived [2]
Indoxyl sulfate	High affinity for both	yes
Indole-3-acetic acid	Moderate affinity for both	yes
Kynurenine	High affinity for both	yes
Kynurenic acid	Moderate affinity for both	yes
p-cresyl sulfate	High affinity for both	yes
p-cresyl glucuronide	No affinity	yes
Hippuric acid	High affinity for both	yes
Phenylacetylglutamine	No affinity	yes
CMPF	More studies are needed to confirm, affinity rather for OAT3)	no
TMAO	Not confirmed yet	yes
Urée	No affinity	no

Figure S1. Distribution of log normalized uremic toxins according to the stages of chronic kidney disease.

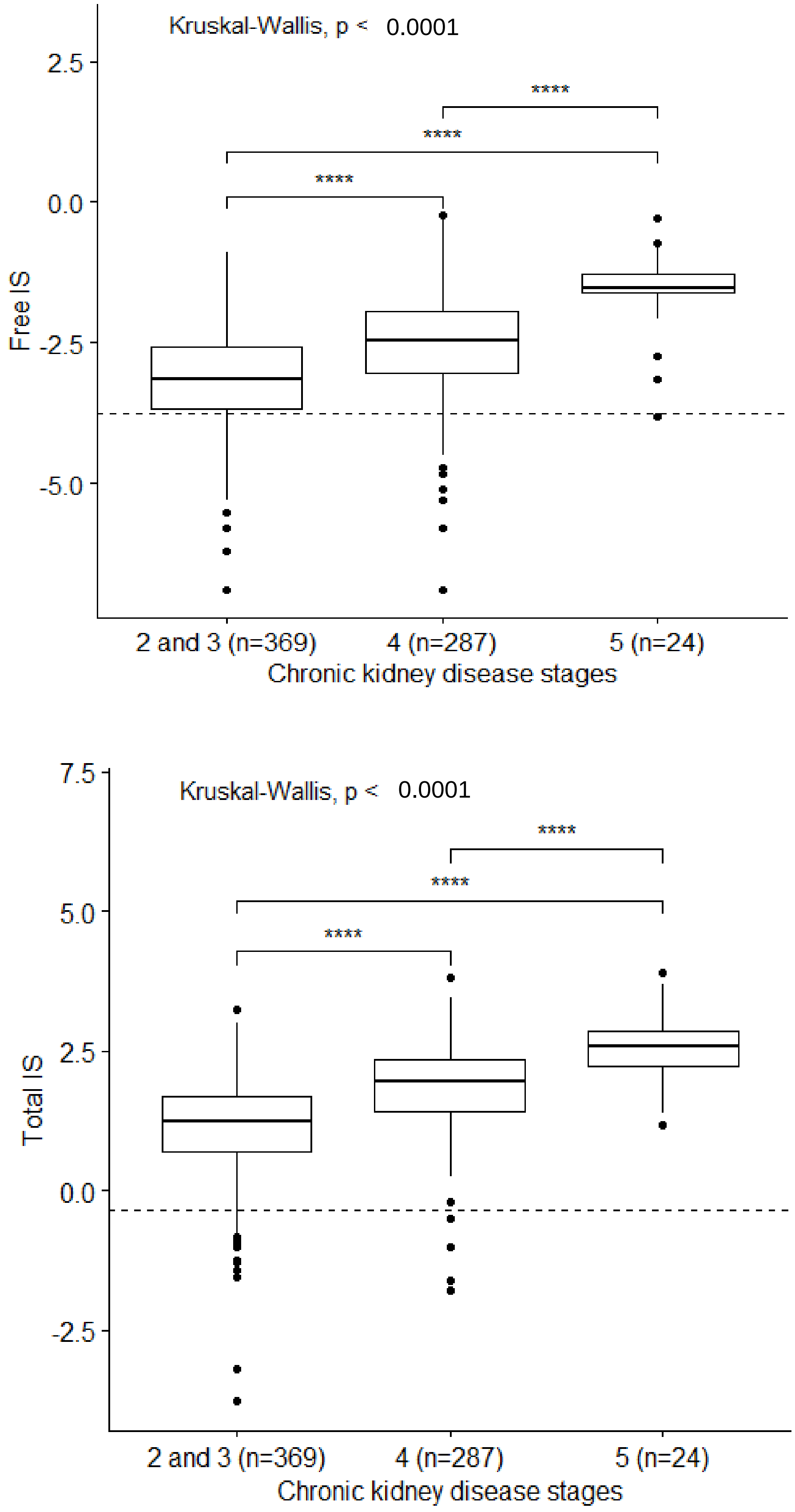

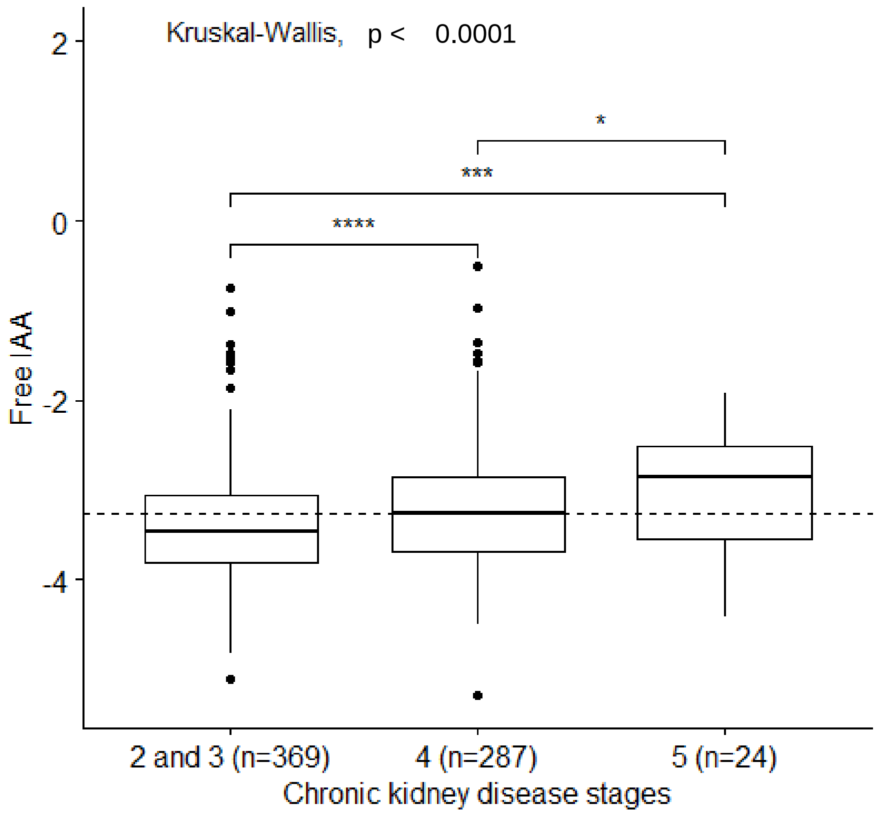
Kruskal-Wallis, p < 0.0001
*
***
****
2
0
-2
-4
Free IAA
2 and 3 (n=369)
4 (n=287)
5 (n=24)
Chronic kidney disease stages

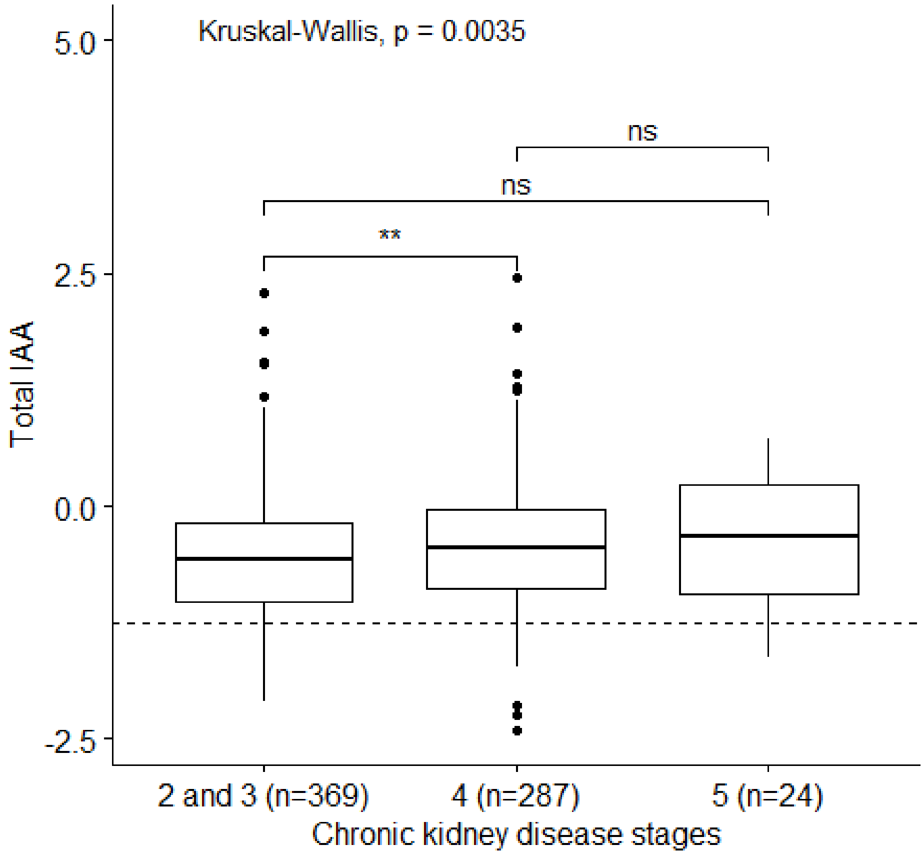
Kruskal-Wallis, p = 0.0035
ns
ns
**
5.0
2.5
0.0
-2.5
Total IAA
2 and 3 (n=369)
4 (n=287)
5 (n=24)
Chronic kidney disease stages

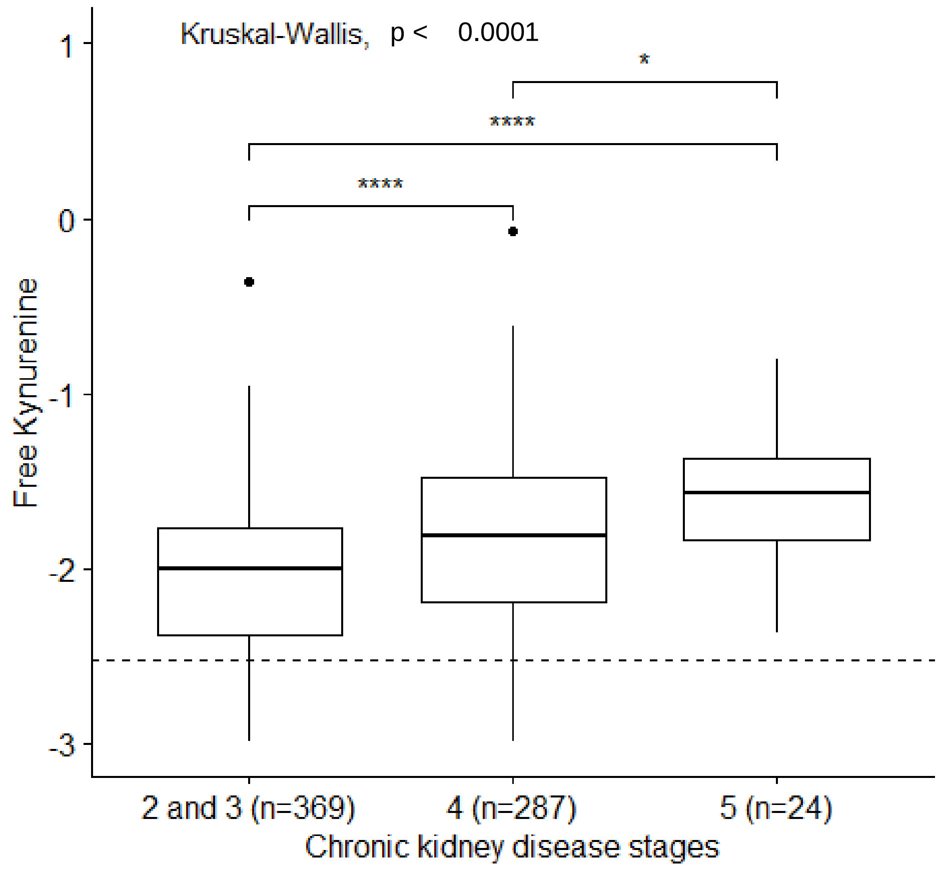
Kruskal-Wallis, p < 0.0001
*
****
****
Free Kynurenine
1
0
-1
-2
-3
2 and 3 (n=369)
4 (n=287)
5 (n=24)
Chronic kidney disease stages

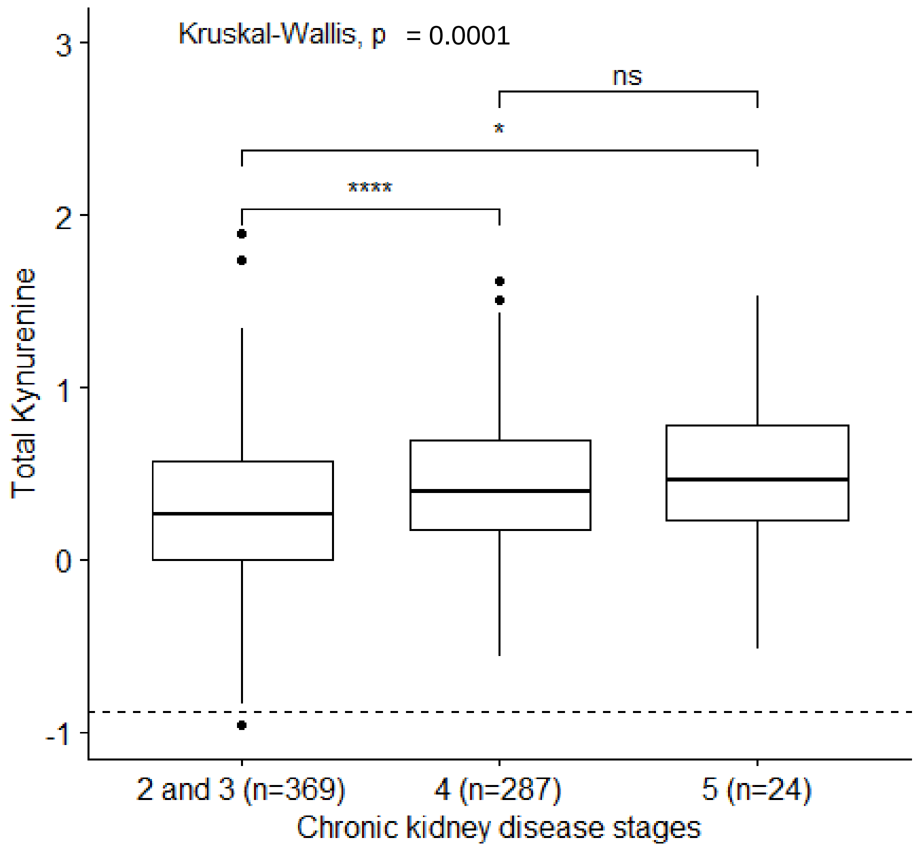
Kruskal-Wallis, p = 0.0001
ns
*
****
Total Kynurenine
3
2
1
0
-1
2 and 3 (n=369)
4 (n=287)
5 (n=24)
Chronic kidney disease stages

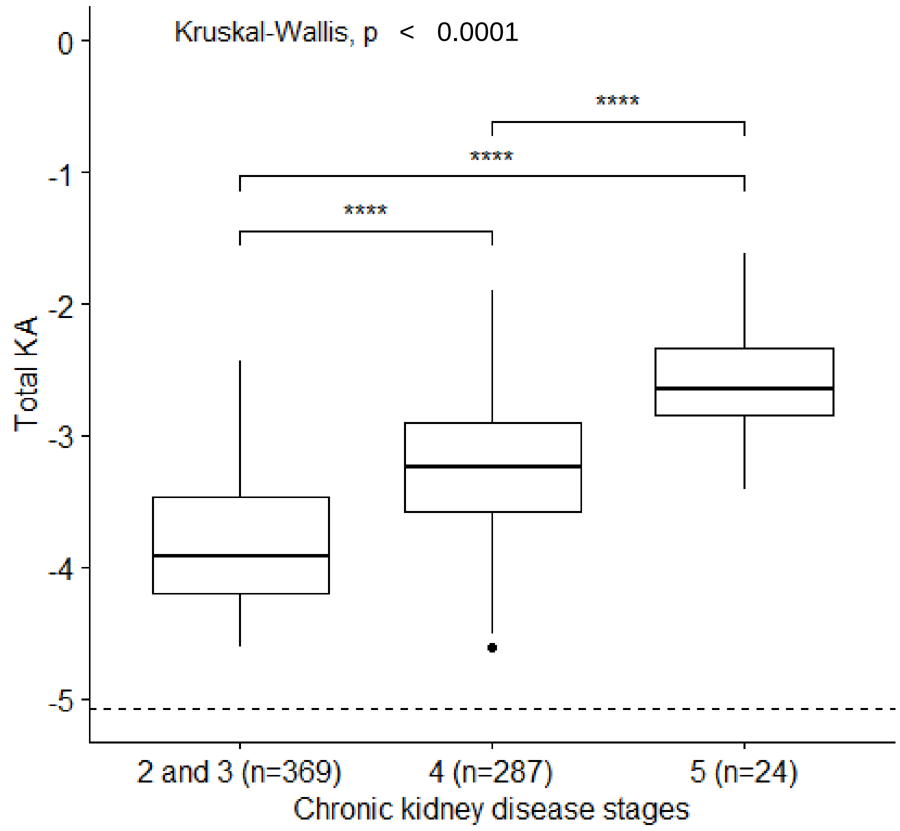
Kruskal-Wallis, p < 0.0001
****
****
****
0
-1
-2
-3
-4
-5
Total KA
2 and 3 (n=369)
4 (n=287)
5 (n=24)
Chronic kidney disease stages

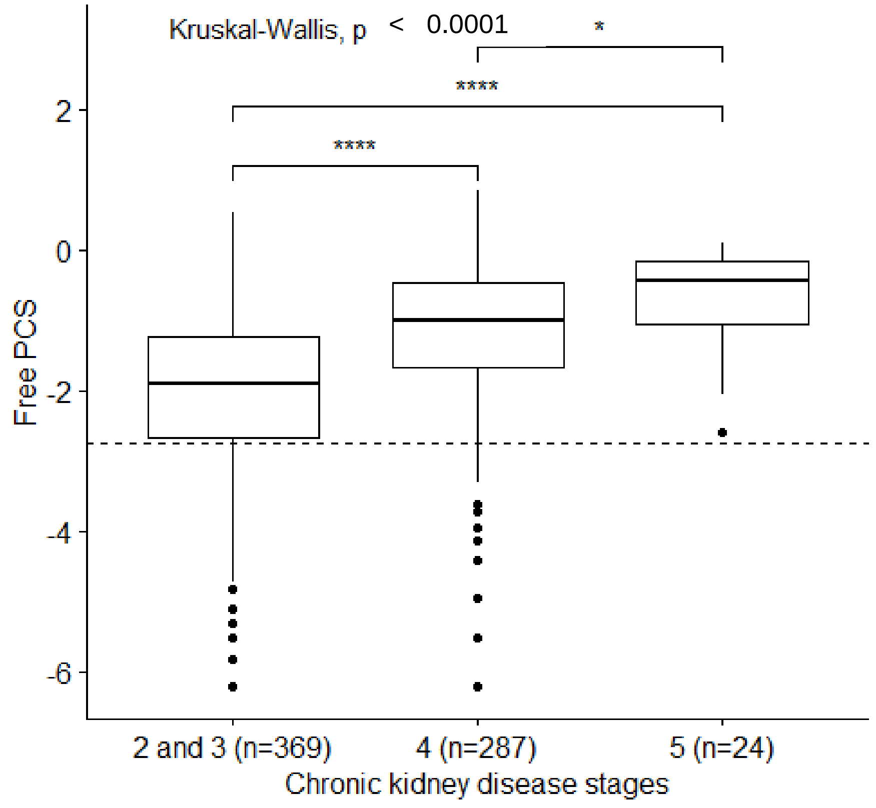
Kruskal-Wallis, p < 0.0001
*
****
****
2
0
-2
-4
-6
Free PCS
2 and 3 (n=369)
4 (n=287)
5 (n=24)
Chronic kidney disease stages

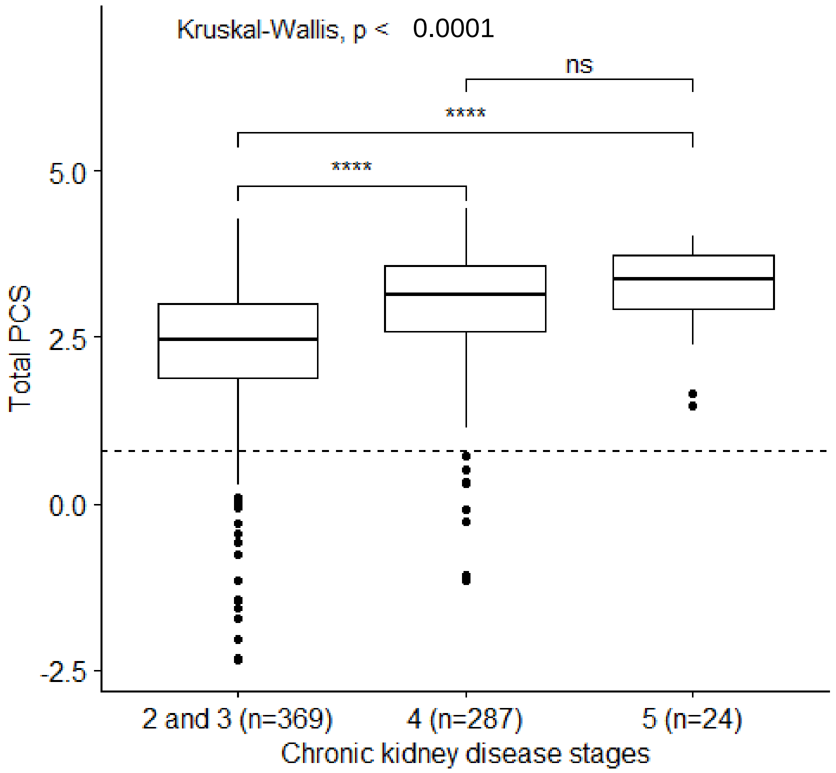
Kruskal-Wallis, p < 0.0001
ns
****
****
Total PCS
5.0
2.5
0.0
-2.5
2 and 3 (n=369)
4 (n=287)
5 (n=24)
Chronic kidney disease stages

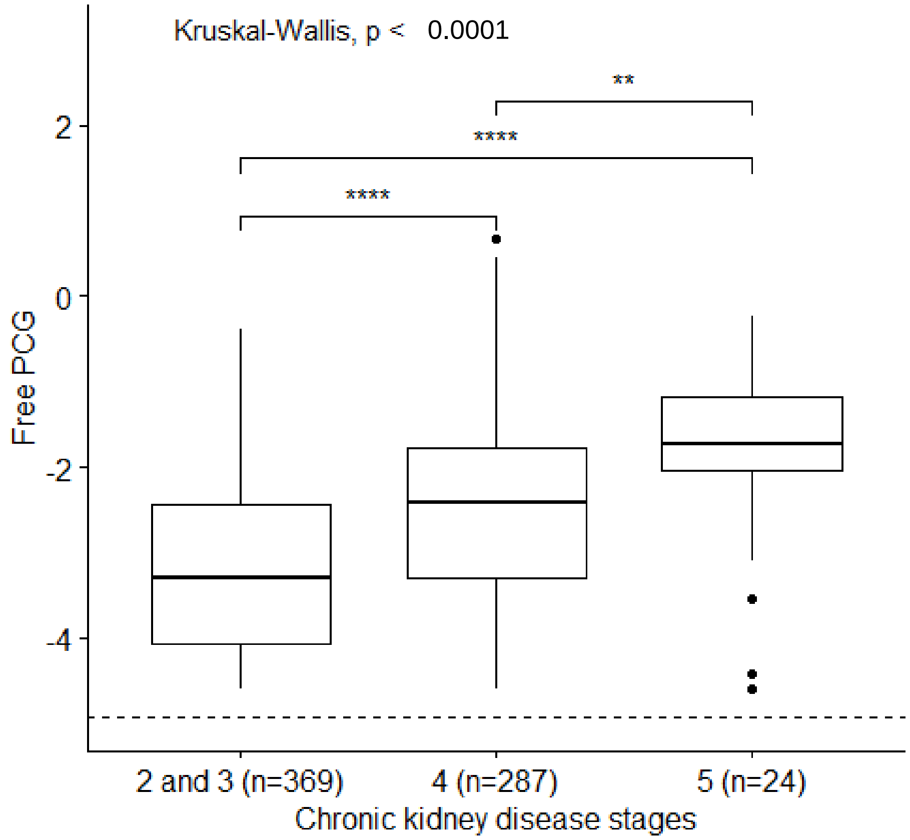
Kruskal-Wallis, p < 0.0001
**
****
****
Free PCG
2
0
-2
-4
2 and 3 (n=369)
4 (n=287)
5 (n=24)
Chronic kidney disease stages

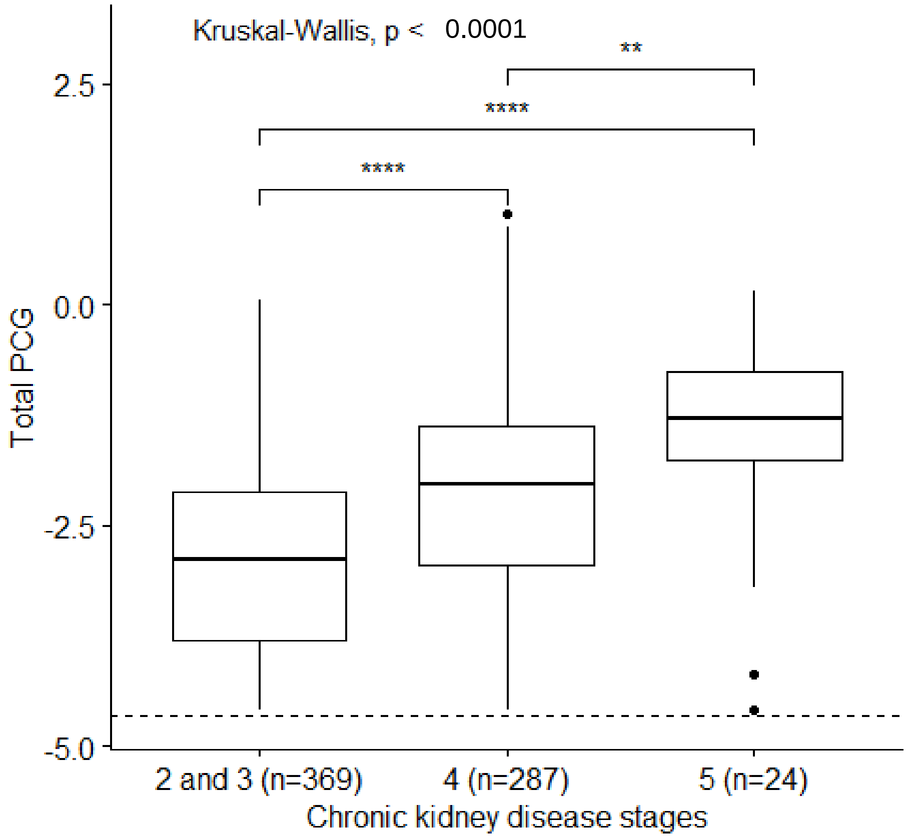
Kruskal-Wallis, p < 0.0001
**
****
****
2.5
0.0
-2.5
-5.0
Total PCG
2 and 3 (n=369)
4 (n=287)
5 (n=24)
Chronic kidney disease stages

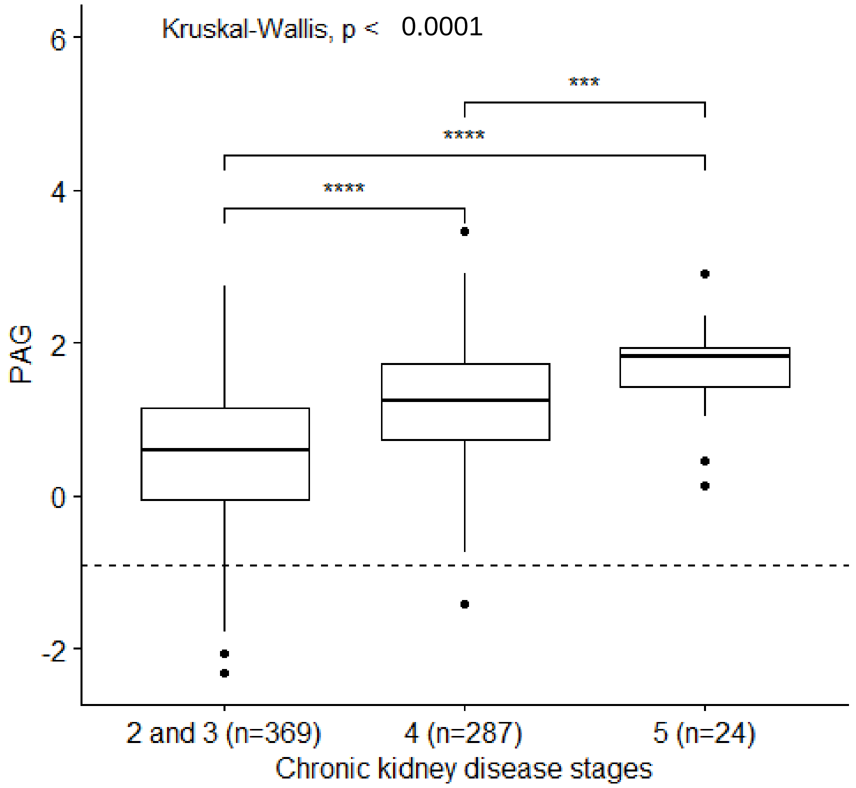
Kruskal-Wallis, p < 0.0001
***
****
****
6
4
2
0
-2
PAG
2 and 3 (n=369)
4 (n=287)
5 (n=24)
Chronic kidney disease stages

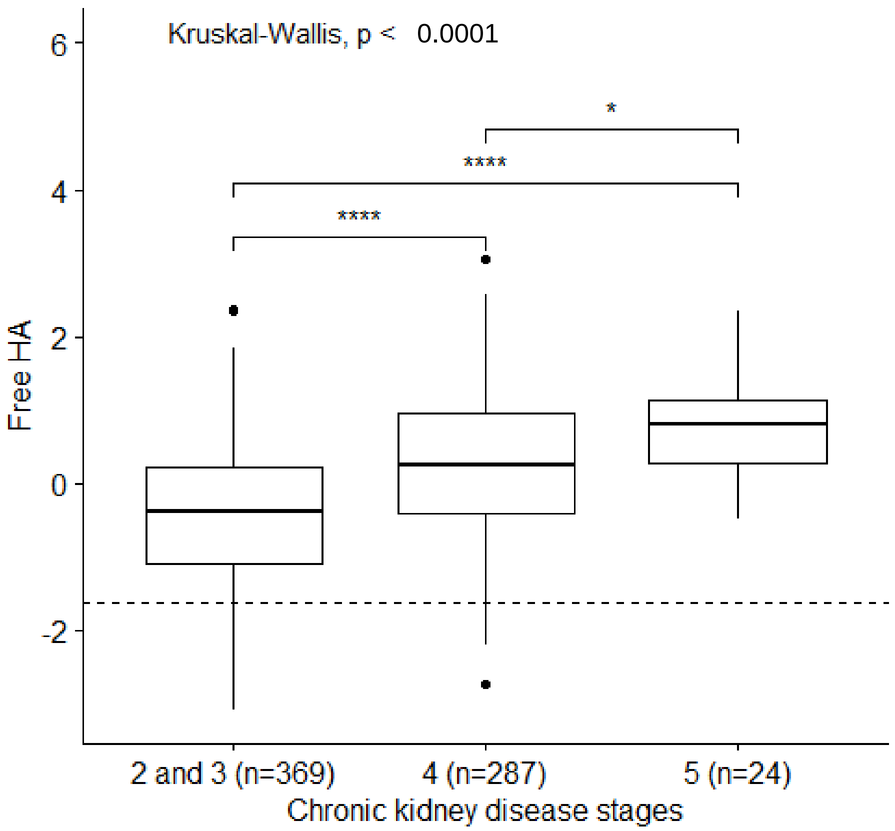
Kruskal-Wallis, p < 0.0001
*
****
****
Free HA
6
4
2
0
-2
2 and 3 (n=369)
4 (n=287)
5 (n=24)
Chronic kidney disease stages

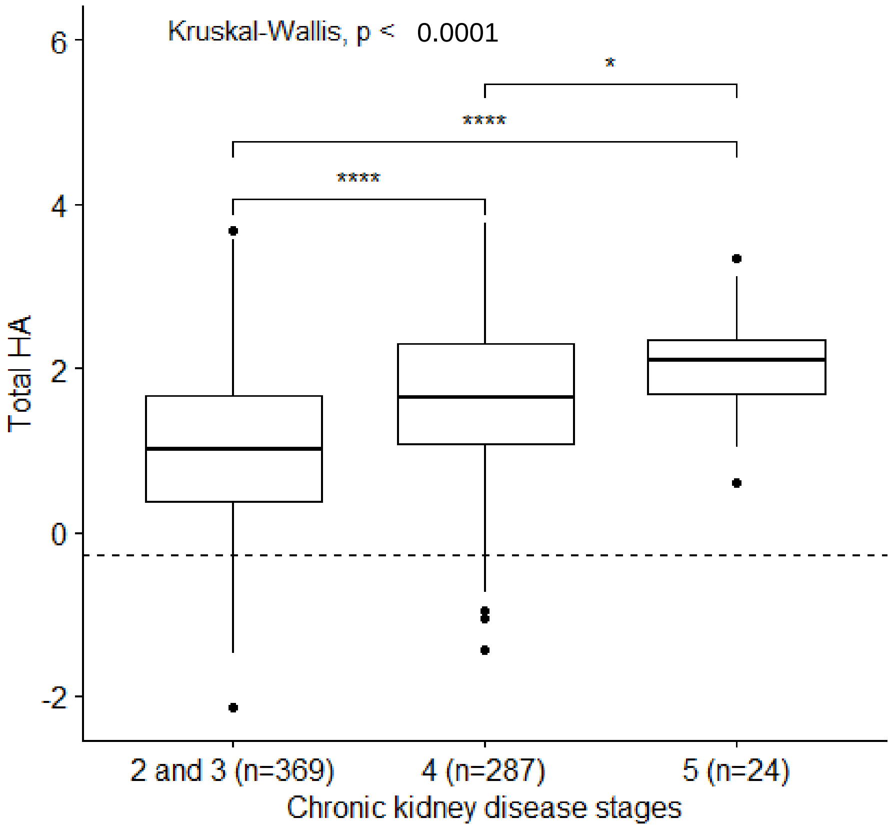
Kruskal-Wallis, p < 0.0001
*
****
****
Total HA
6
4
2
0
-2
2 and 3 (n=369)
4 (n=287)
5 (n=24)
Chronic kidney disease stages

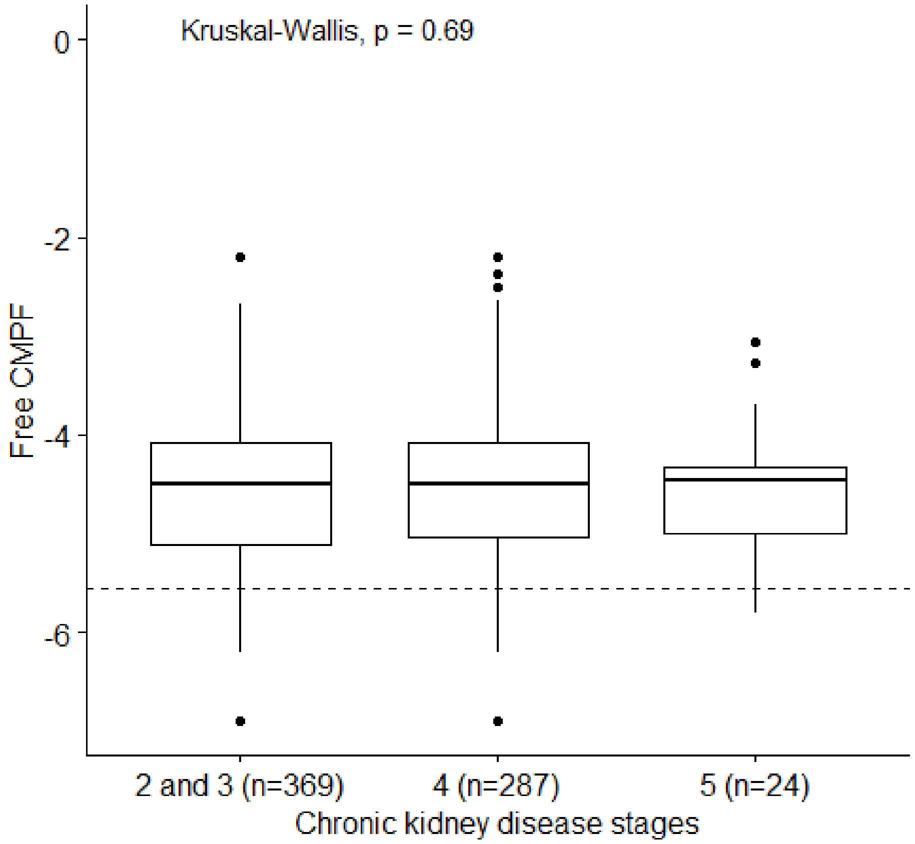
Kruskal-Wallis, p = 0.69
0
-2
-4
-6
Free CMPF
2 and 3 (n=369)
4 (n=287)
5 (n=24)
Chronic kidney disease stages

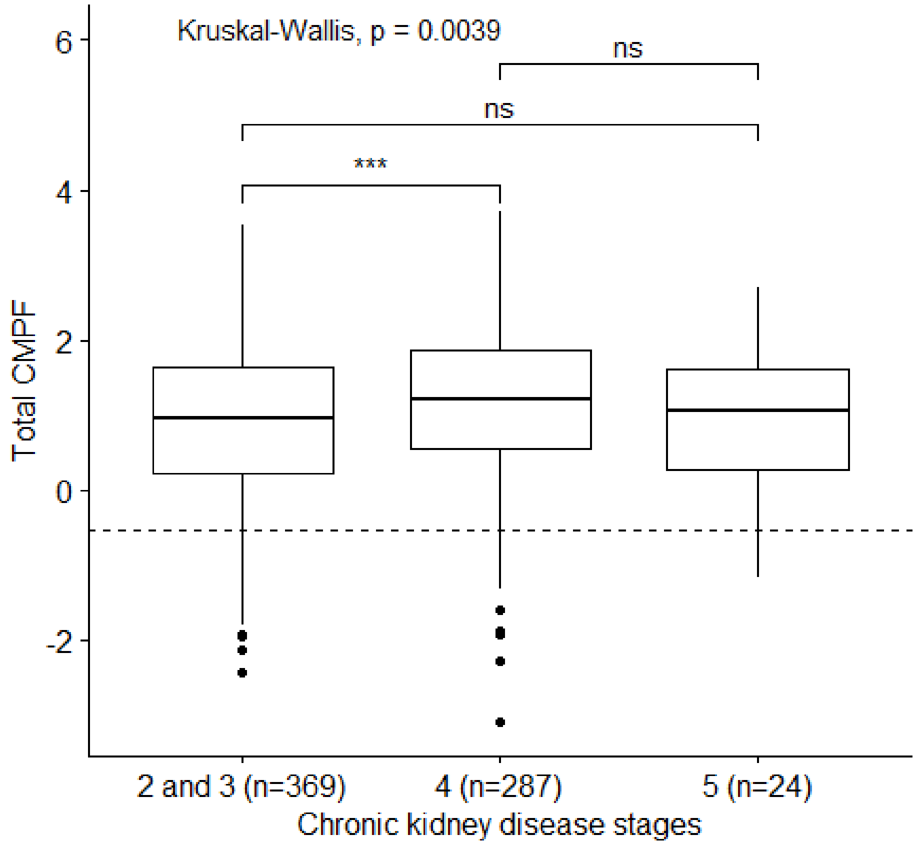
Kruskal-Wallis, p = 0.0039
ns
ns
***
6
4
2
0
-2
Total CMPF
2 and 3 (n=369)
4 (n=287)
5 (n=24)
Chronic kidney disease stages

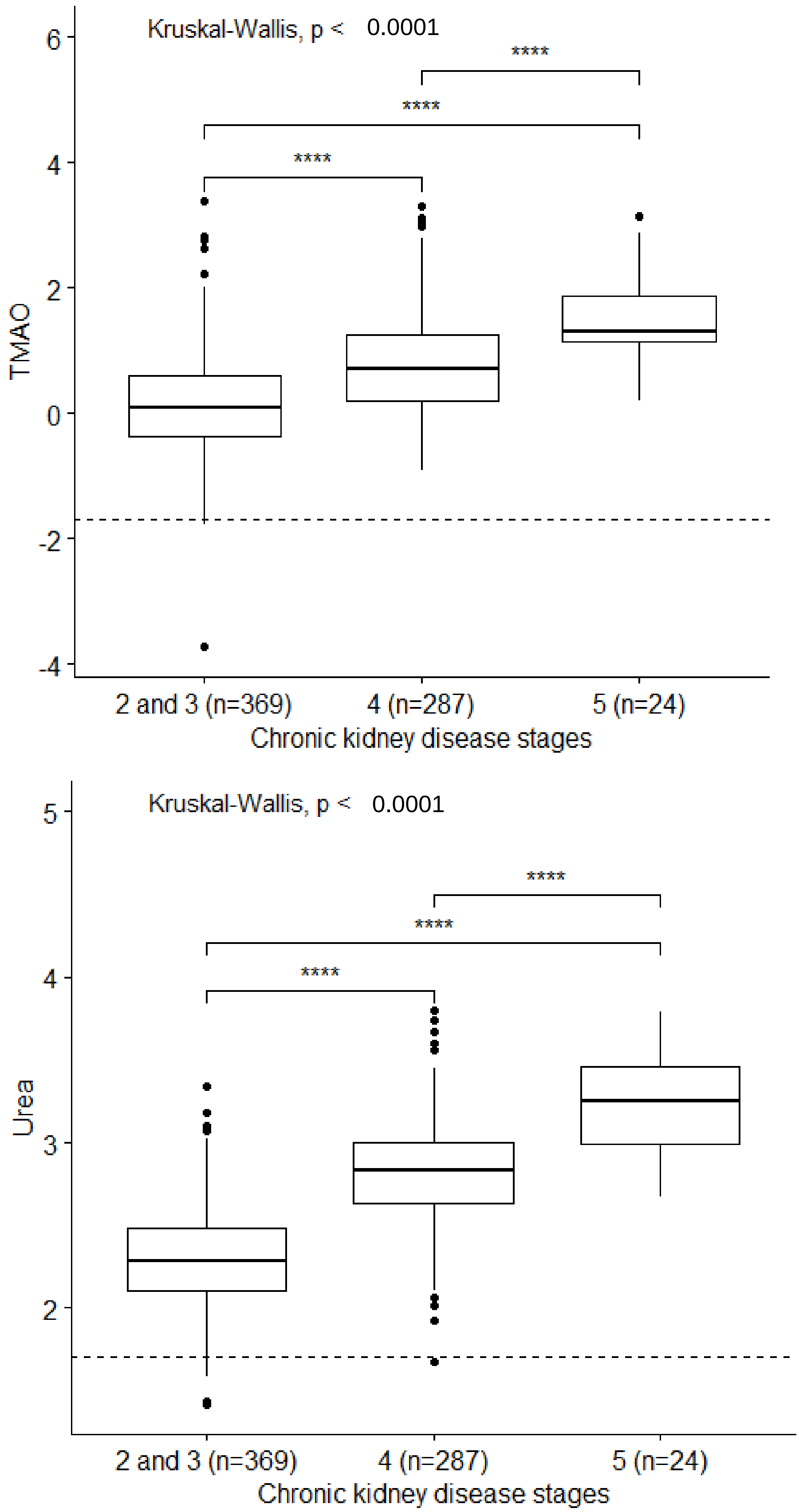

The dotted line indicates the reference value for healthy subjects.
P-values : ns : non significant ; * : <0.05 ; ** : <0.01 ; *** :<0.001 ; **** : <0.0001.
Abbreviations : IS, indoxyl sulfate ; IAA, indole-3-acetic acid ; KA, kynurenic acid ; PCS, p-cresyl sulfate ; PCG, p-cresyl glucuronide ; PAG, phenylacetylglutamine ; HA, hippuric acid ; CMPF, 3-carboxy-4-ethyl-5-propyl-2-furanpropanoic acid ; TMAO, trimethylamine N-oxide.

Figure S2. Distribution of log normalized uremic toxins according to proton-pupm inhibitor use.

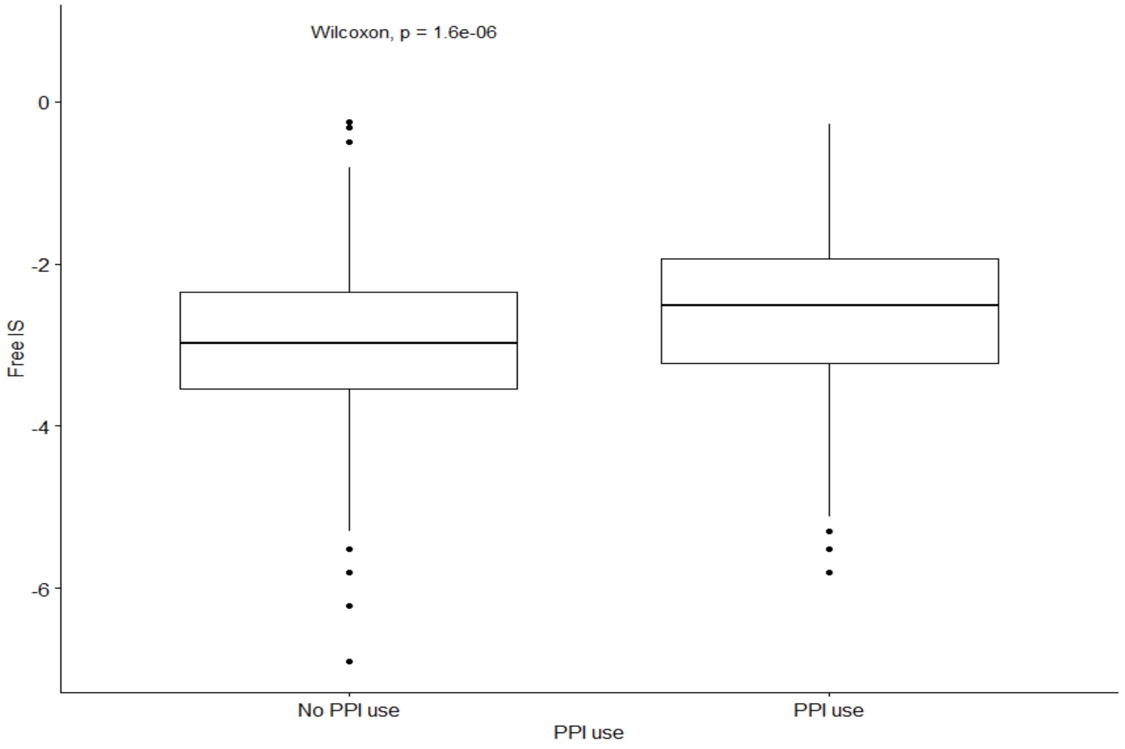

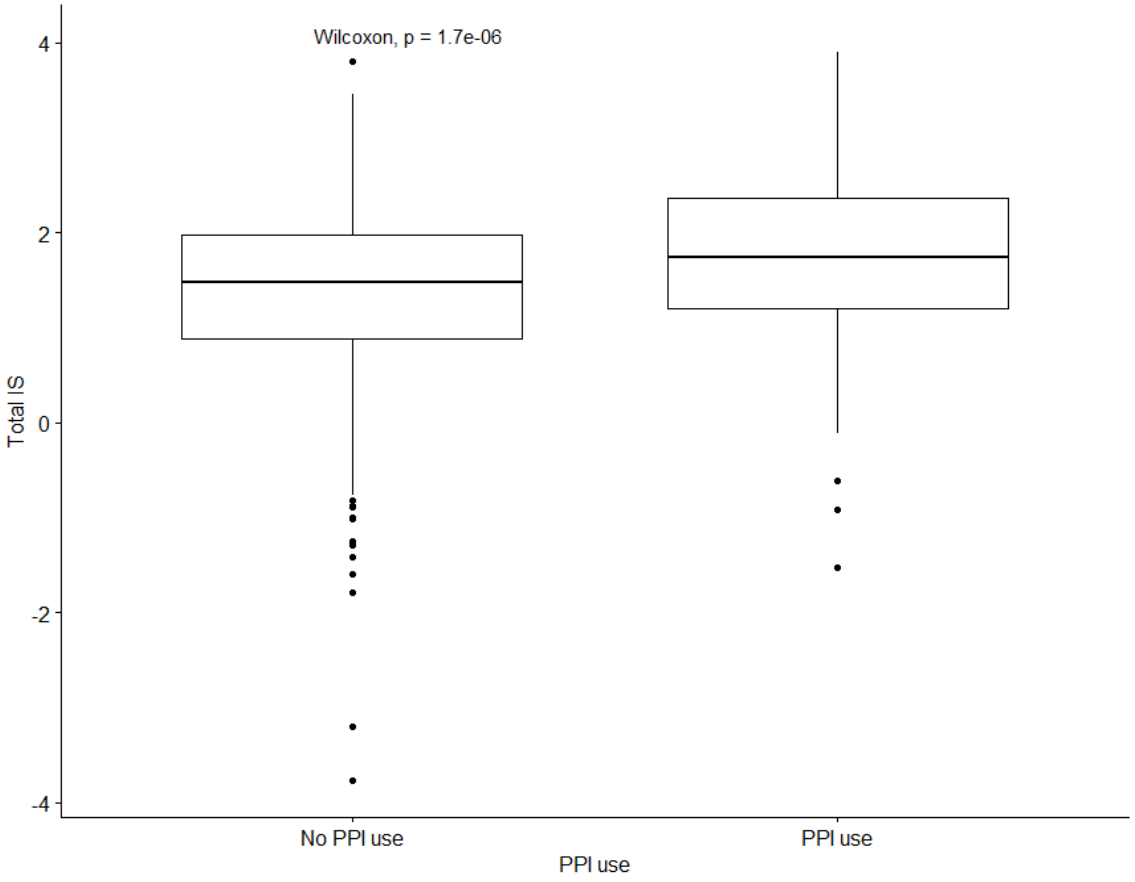

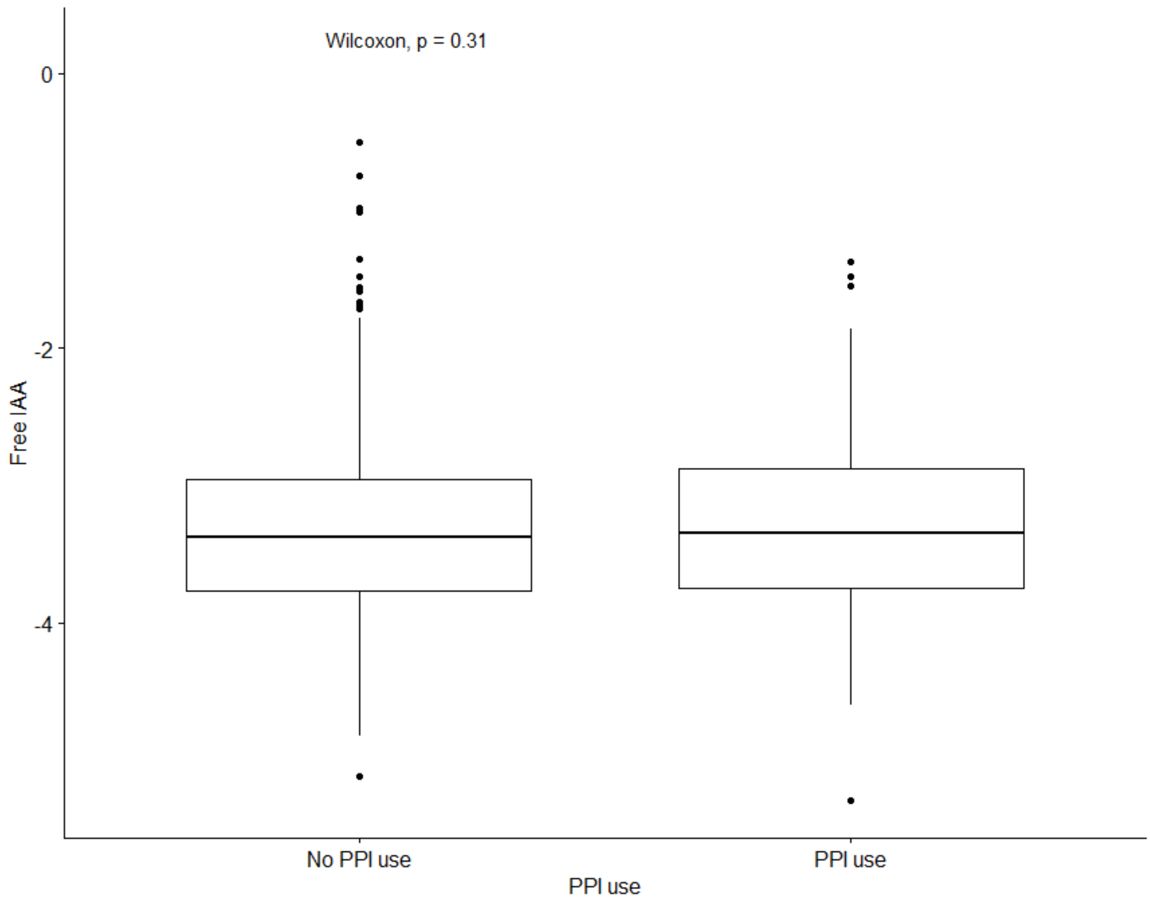

Wilcoxon, p = 0.31
0
-2
-4
Free IAA
No PPI use
PPI use
PPI use

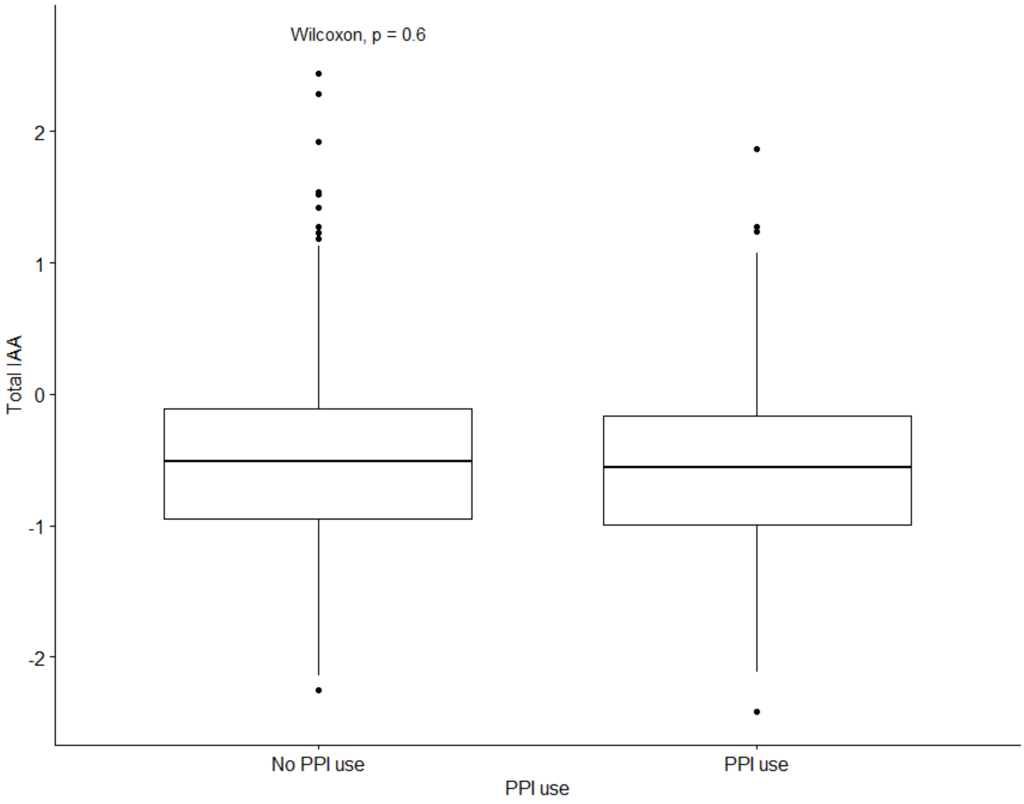

Wilcoxon, p = 0.6
2
1
0
-1
-2
Total IAA
No PPI use
PPI use
PPI use

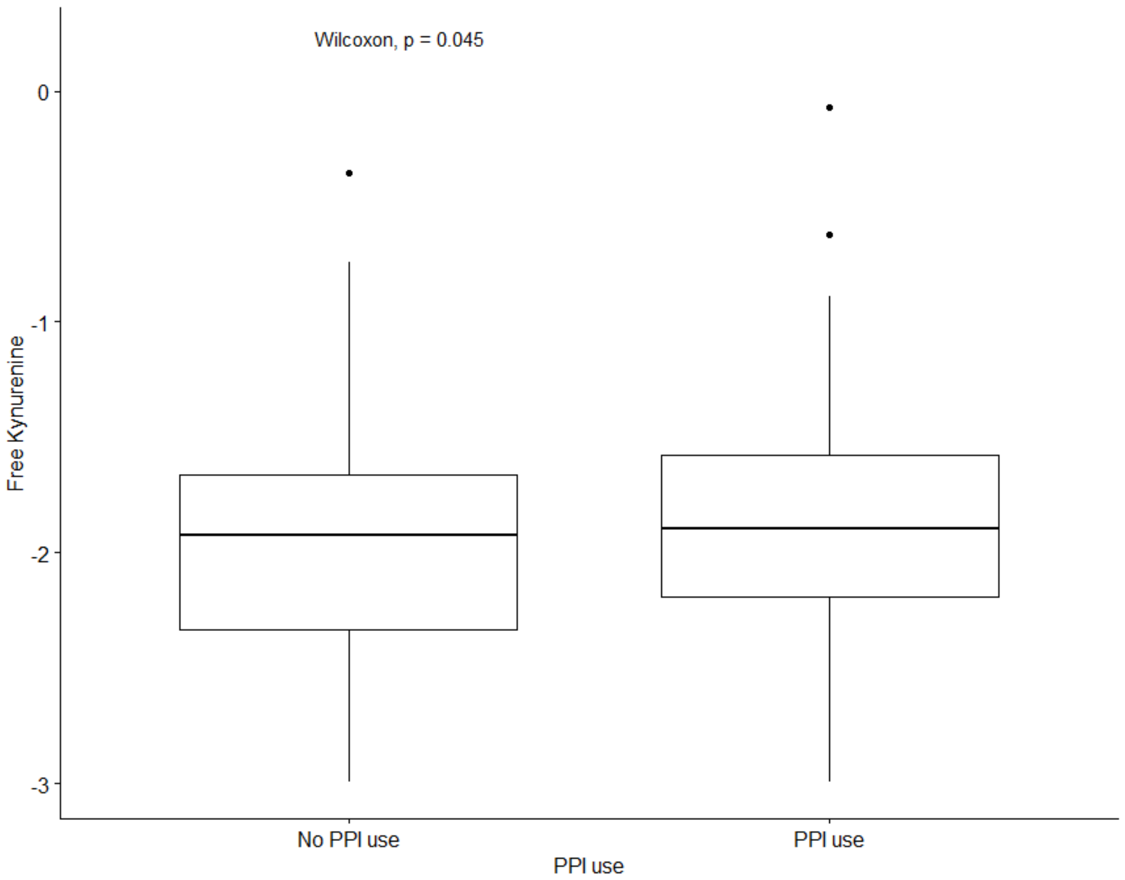
Wilcoxon, p = 0.045
0
-1
-2
-3
Free Kynurenine
No PPI use
PPI use
PPI use

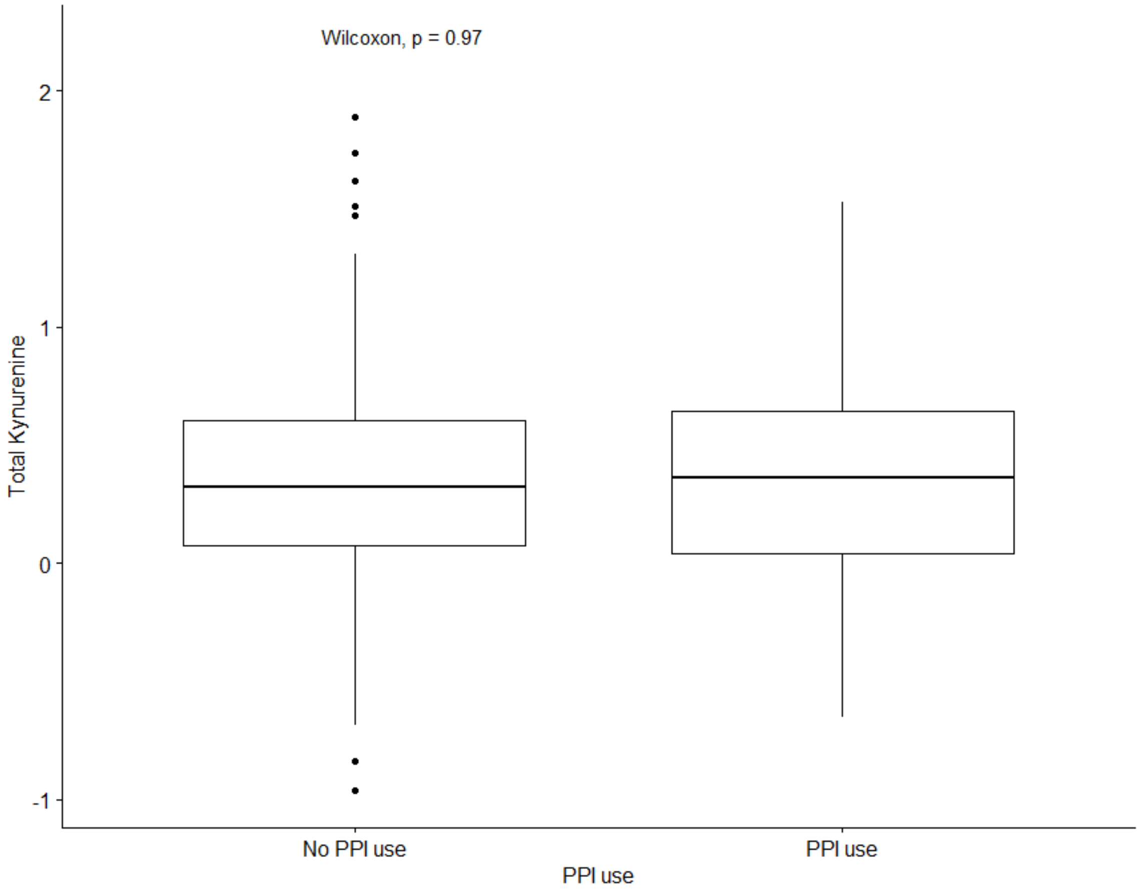
Wilcoxon, p = 0.97
2
1
0
-1
Total Kynurenine
No PPI use
PPI use
PPI use

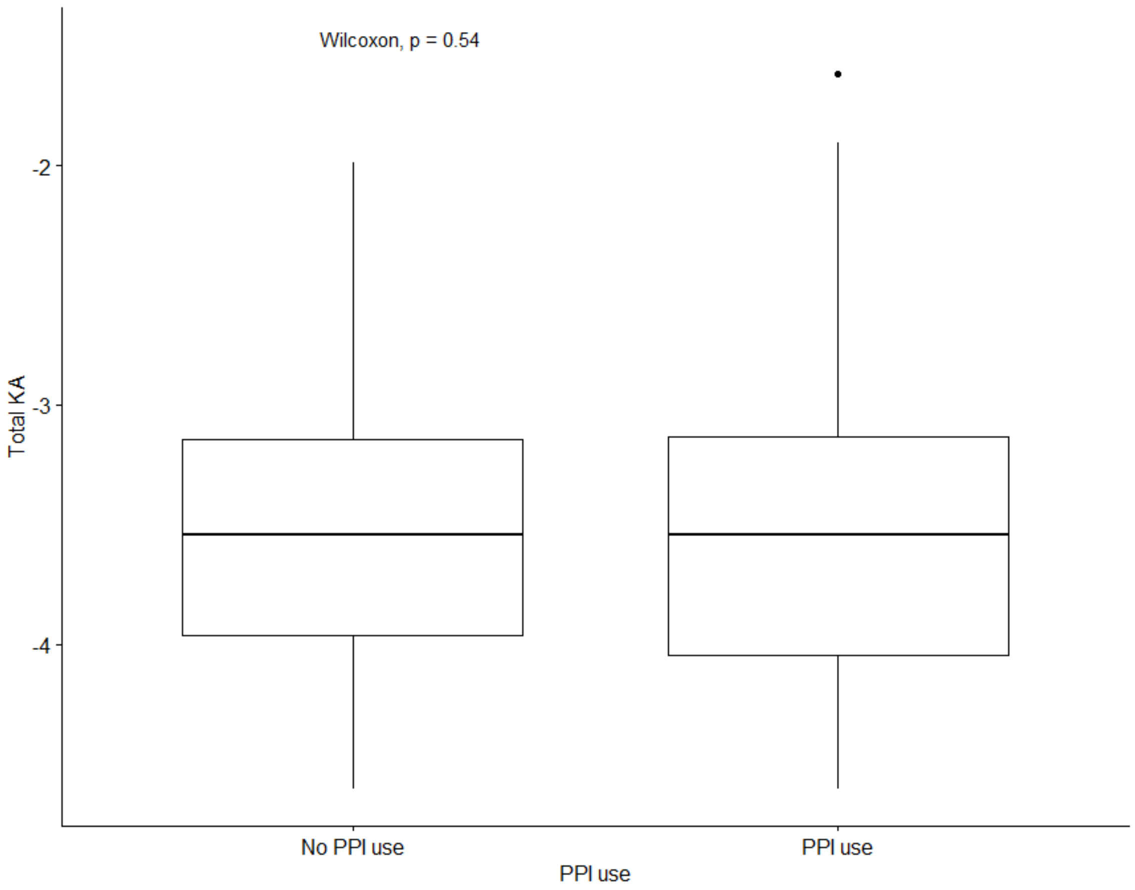
Wilcoxon, p = 0.54
Total KA
-2
-3
-4
No PPI use
PPI use
PPI use

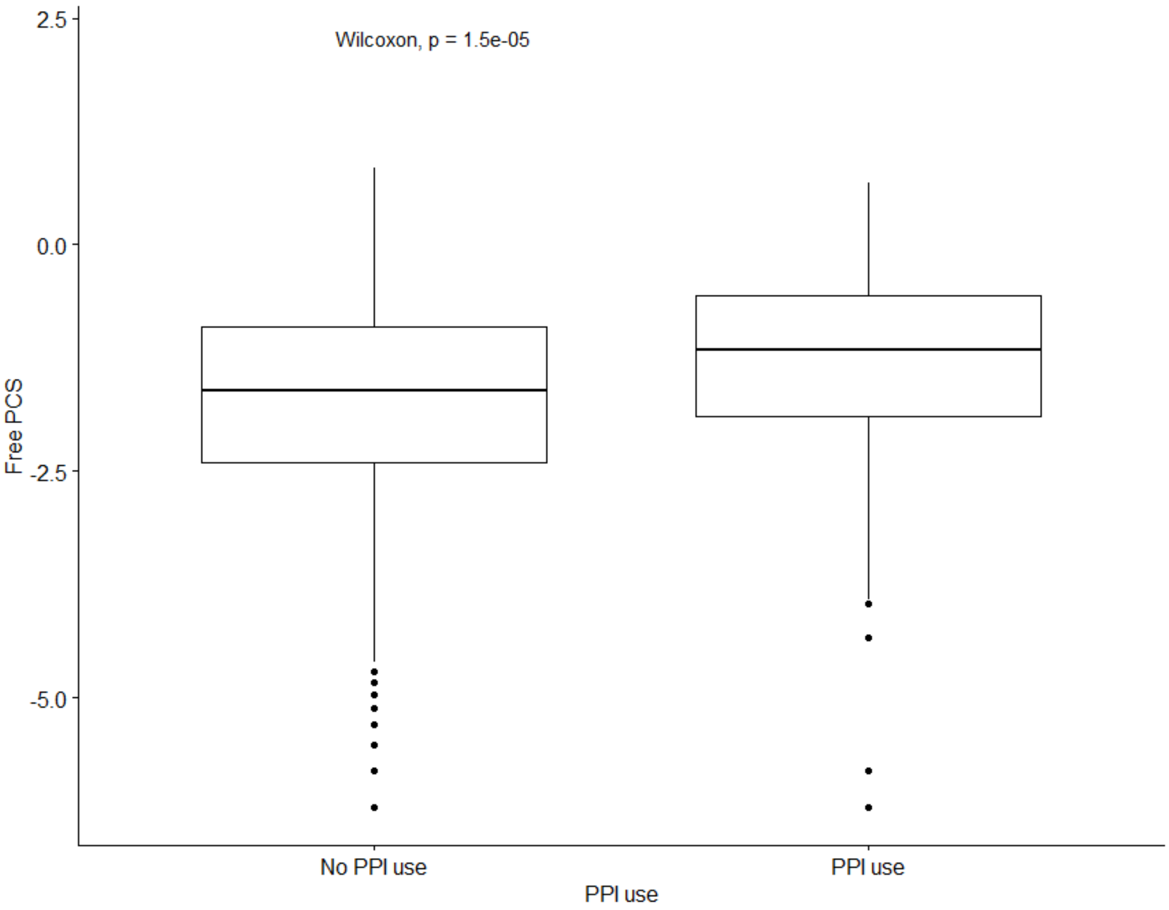
Wilcoxon, p = 1.5e-05
Free PCS
2.5
0.0
-2.5
-5.0
No PPI use
PPI use
PPI use

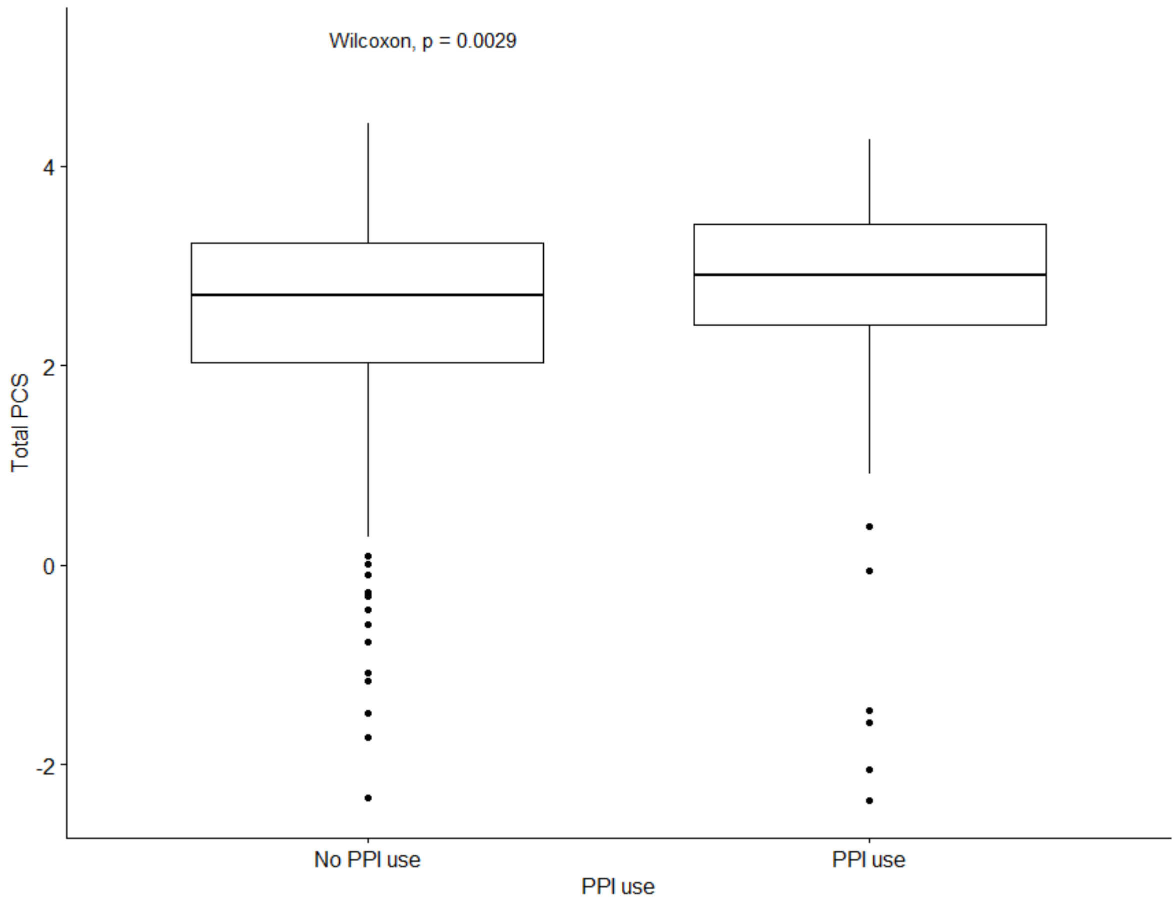
Wilcoxon, p = 0.0029
Total PCS
4
2
0
-2
No PPI use
PPI use
PPI use

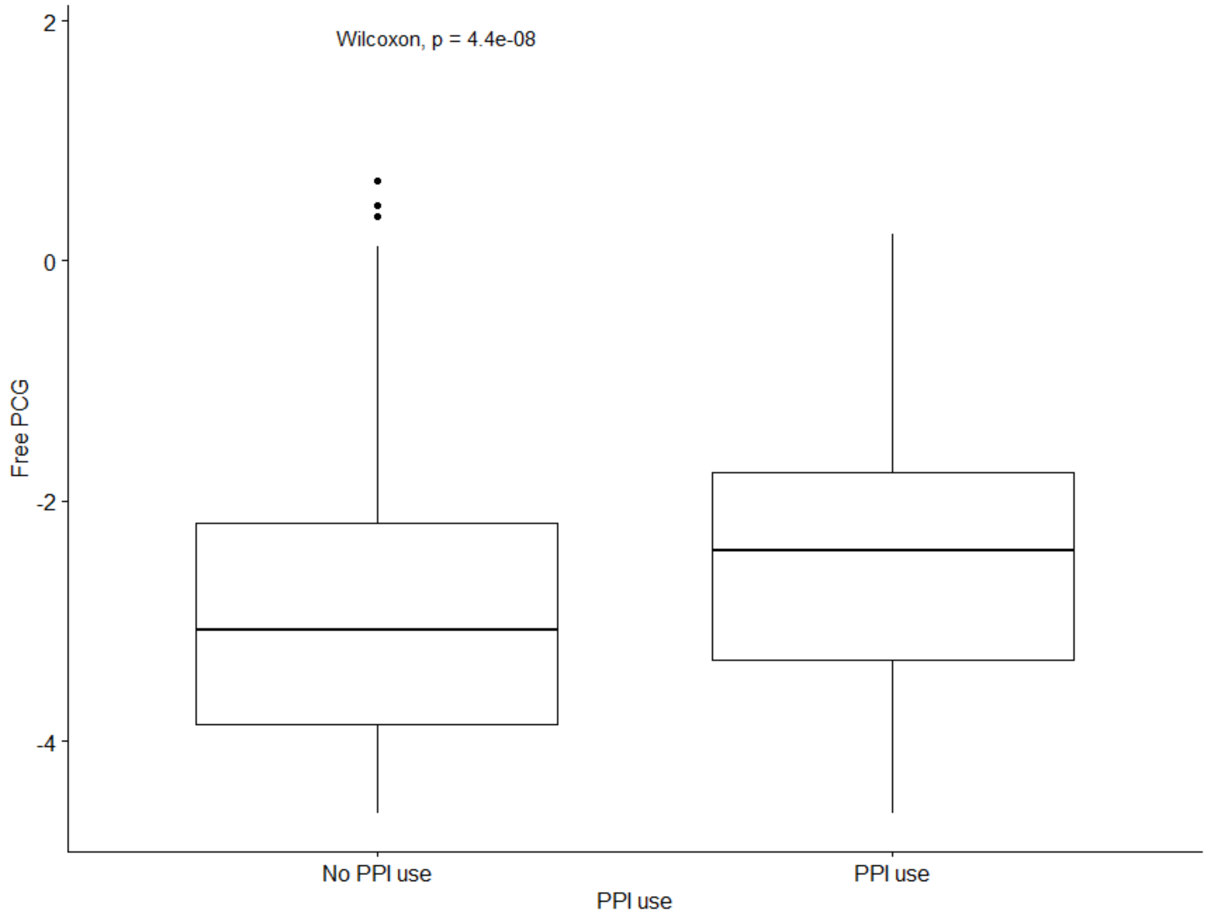
Wilcoxon, p = 4.4e-08
Free PCG
2
0
-2
-4
No PPI use
PPI use
PPI use

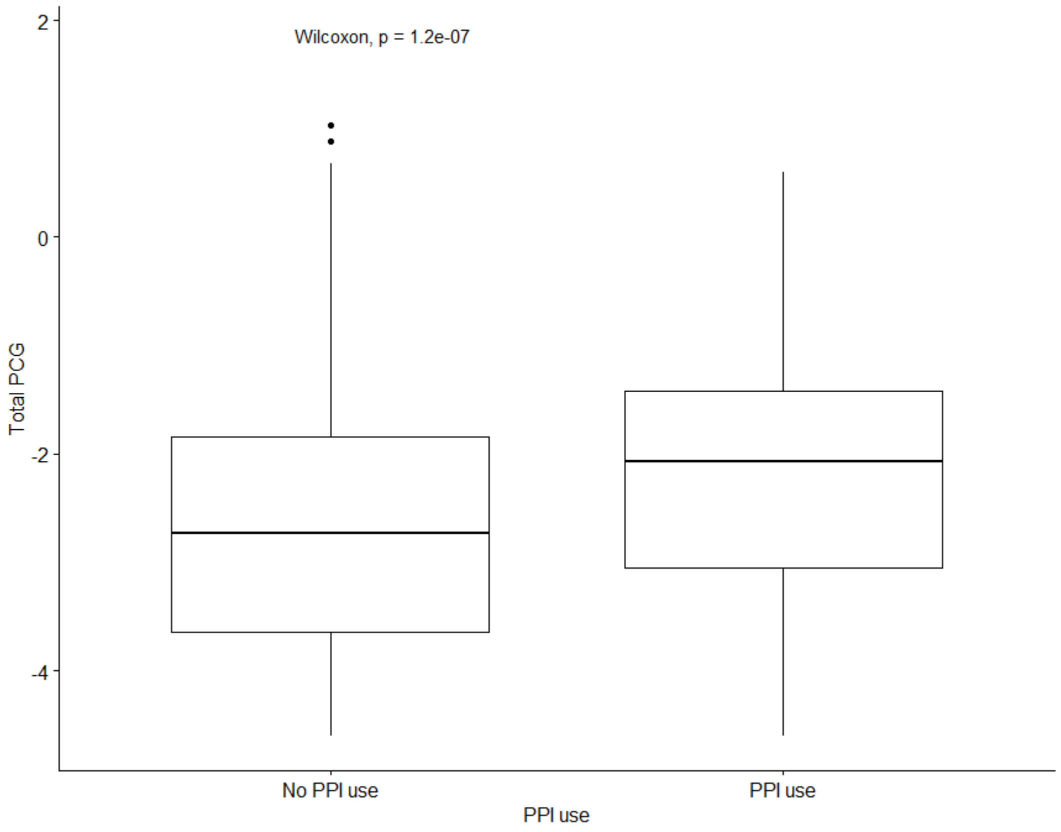
Wilcoxon, p = 1.2e-07
Total PCG
2
0
-2
-4
No PPI use
PPI use
PPI use

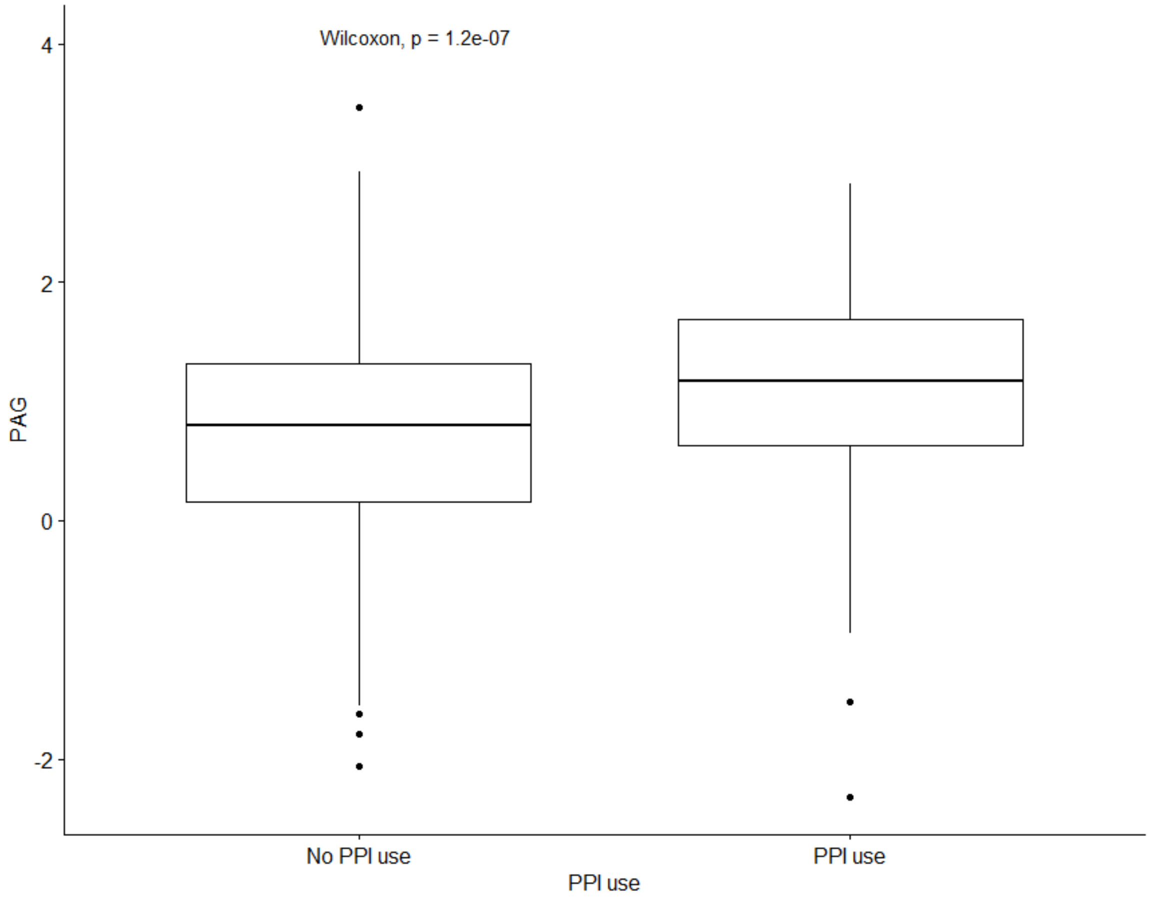
Wilcoxon, p = 1.2e-07
PAG
4
2
0
-2
No PPI use
PPI use
PPI use

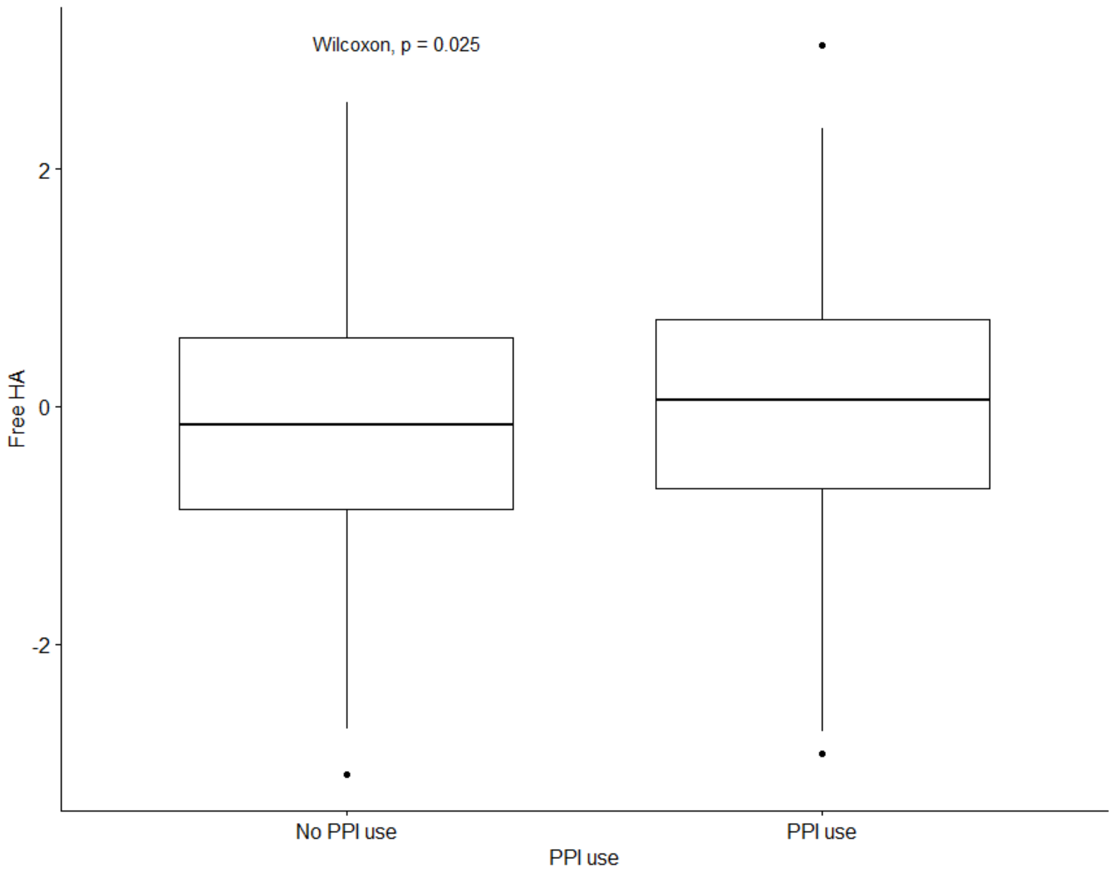
Wilcoxon, p = 0.025
2
0
-2
Free HA
No PPI use
PPI use
PPI use

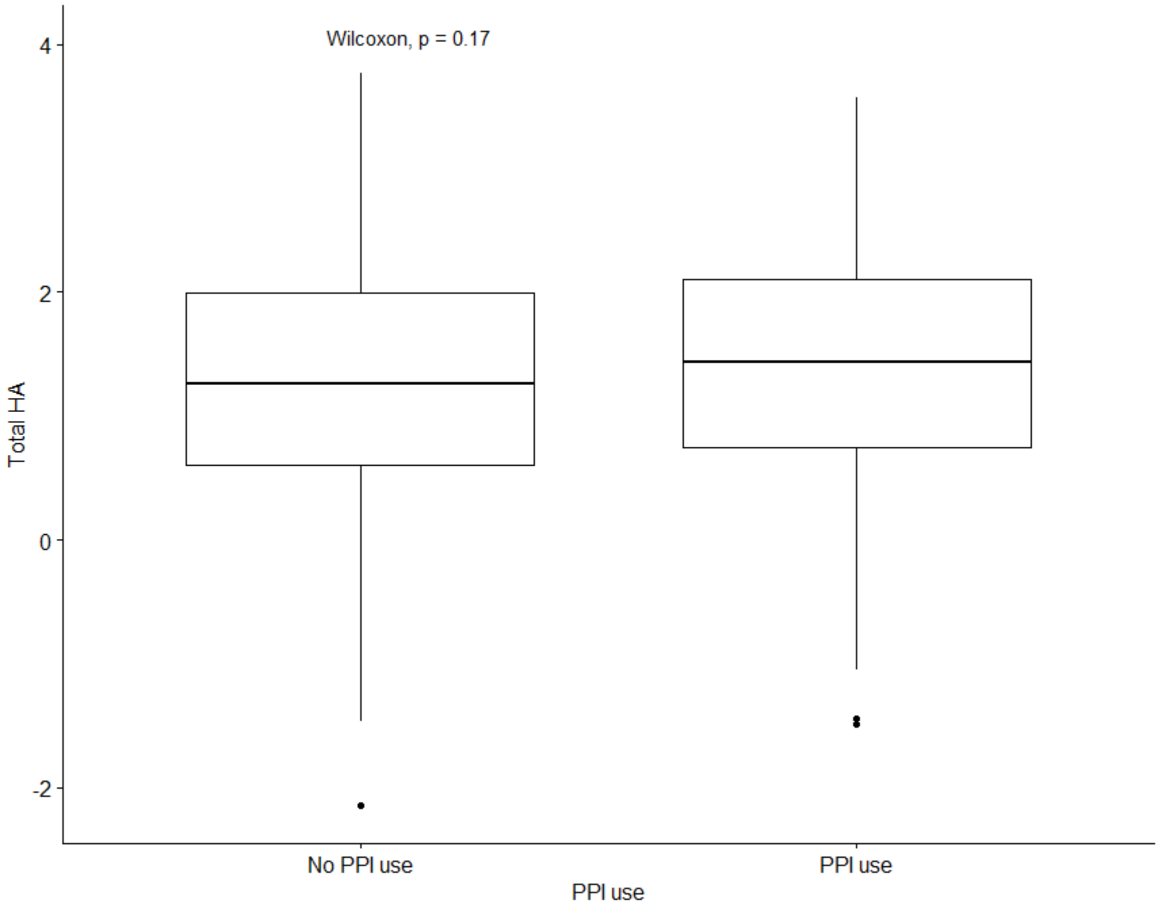
Wilcoxon, p = 0.17
4
2
0
-2
Total HA
No PPI use
PPI use
PPI use

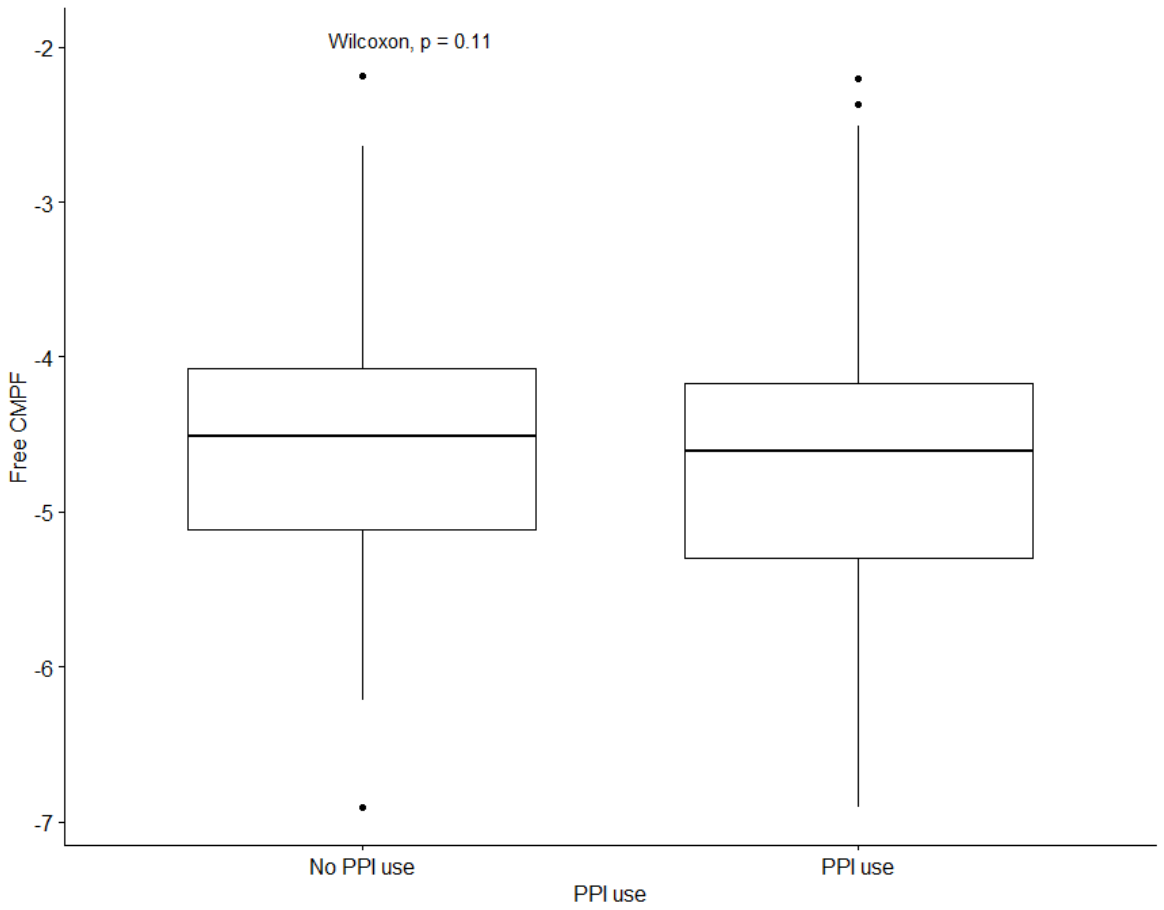
Wilcoxon, p = 0.11
-2
-3
-4
-5
-6
-7
Free CMPF
No PPI use
PPI use
PPI use

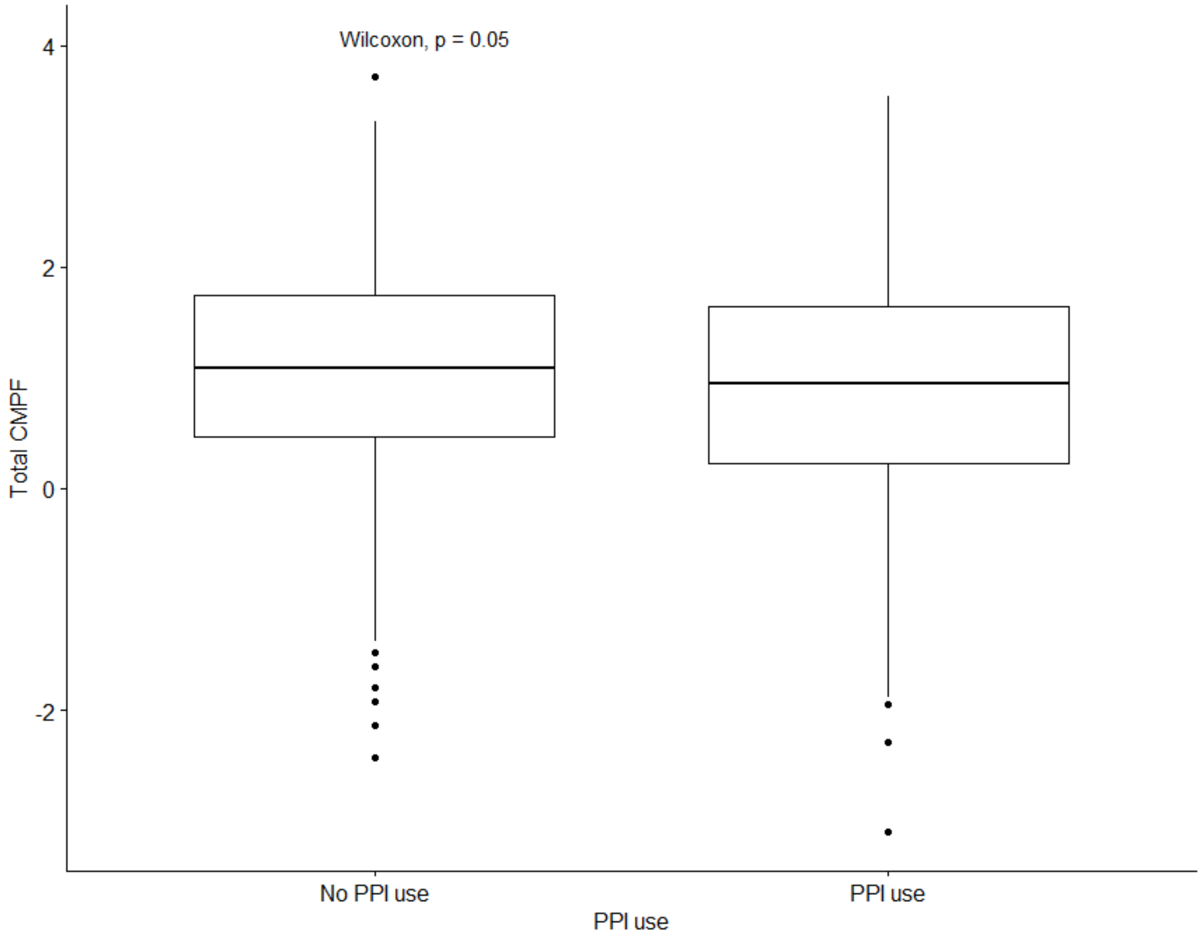
Wilcoxon, p = 0.05
4
2
0
-2
Total CMPF
No PPI use
PPI use
PPI use

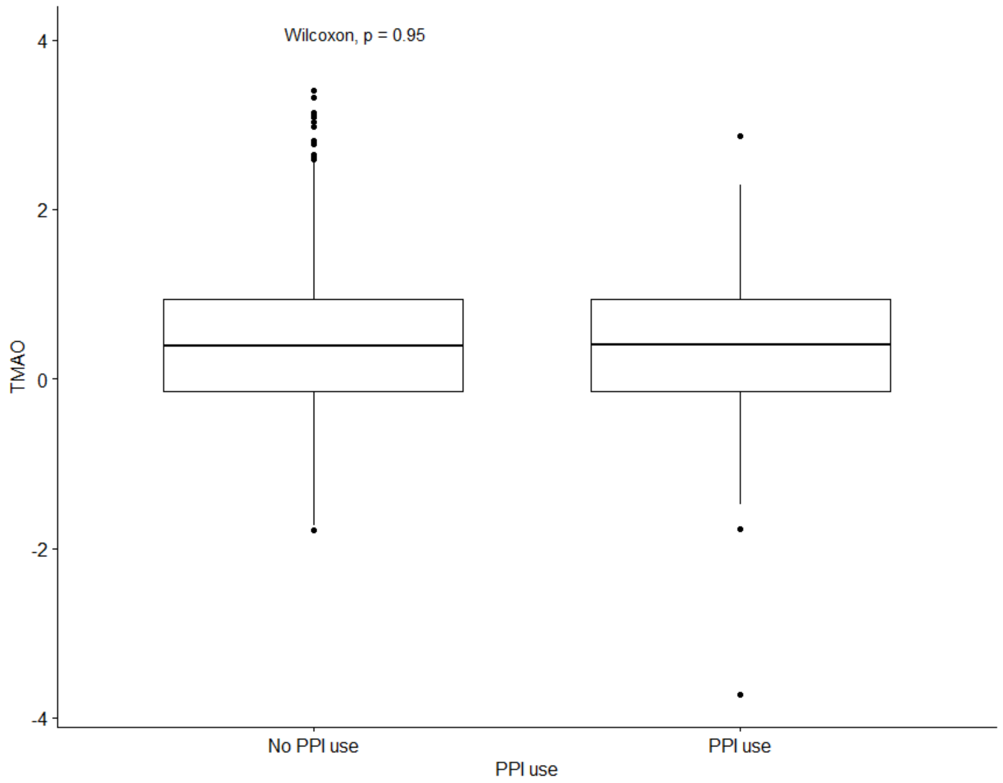

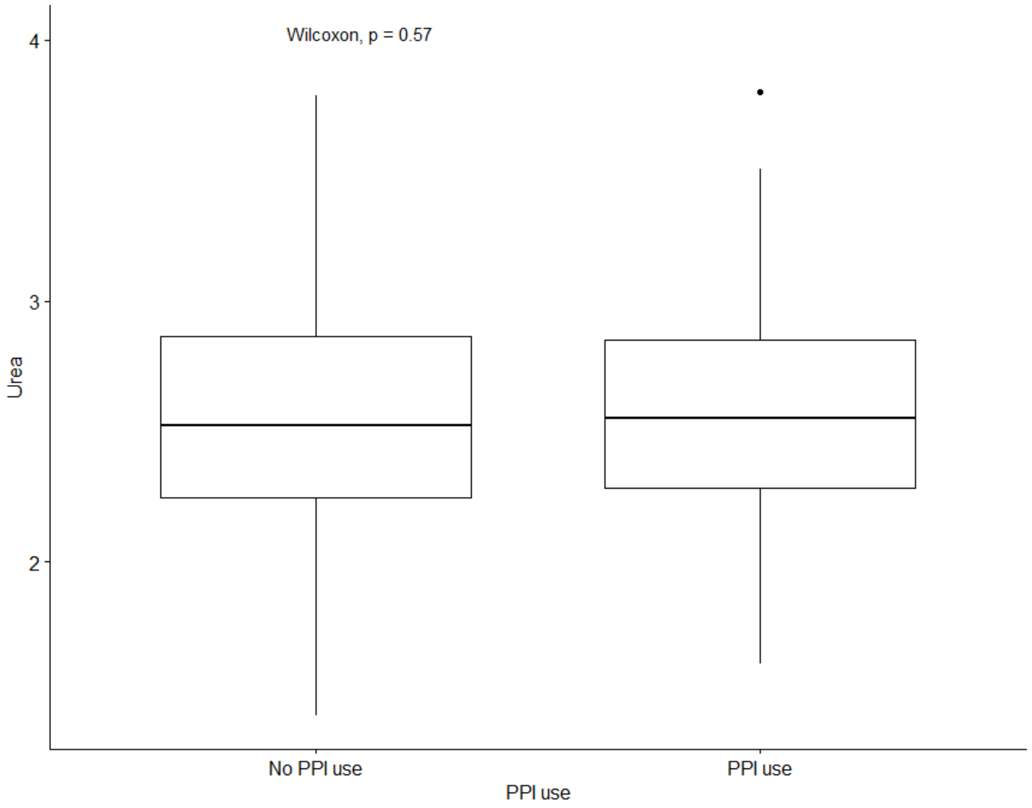

Abbreviations : PPI : proton pump inhibitor ; IS, indoxyl sulfate ; IAA, indole-3-acetic acid ; KA, kynurenic acid ; PCS, p-cresyl sulfate ; PCG, p-cresyl glucuronide ; PAG, phenylacetylglutamine ; HA, hippuric acid ; CMPF, 3-carboxy-4-ethyl-5-propyl-2-furanpropanoic acid ; TMAO, trimethylamine N-oxide.

List of biological resources centers

The authors would like to thank the teams of all the biological resources centers that participated in the CKD-REIN project:

- Biobanque de Picardie, CRB du Centre Hospitalier Universitaire Amiens Picardie, 1 Rond-Point du Pr Christian Cabrol, 80054 Amiens Cedex 1 (BRIF number: BB-0033-00017)
- NeuroBioTec, CRB des Hospices Civils de Lyon Groupement Hospitalier Est, Hôpital Neurologique, 59 Boulevard Pinel, 69677 Bron Cedex (BRIF number: BB-0033-00046)
- Centre de ressources biologiques du Centre Hospitalier Universitaire deNantes Hôtel Dieu, Institut de biologie, 9, quai Moncousu, 44093 Nantes Cedex 1 (BRIF number: BB-0033-00040)
- Centre de ressources biologiques du Centre Hospitalier Universitaire Grenoble Alpes, Boulevard de la Chantourne, CS 10217, 38700 La Tronche (BRIF number: BB-0033-00069)
- Centre de ressources biologiques du Centre Hospitalier Régional Universitaire de Nancy, Hôpitaux de Brabois, Bâtiment Recherche Rue du Morvan, 54500 Vandoeuvre-les-Nancy (BRIF number: BB-0033-00035)
- Servicer de Néphrologie, Centre Hospitalier de Perpignan, 20 Avenue du Languedoc, 66046 Perpignan Cedex 9
- Plateforme de Ressources Biologiques, Hôpital Henri Mondor, 51 avenue du Maréchal de Lattre de Tassigny, 94000 Créteil (BRIF number: BB-0033-00021)
- CIC-1435, Centre d'Investigation Clinique Plurithématique, Centre Hospitalier Universitaire de Limoges, 2 Avenue Martin Luther King, 87042 Limoges Cedex
- Plateforme de Ressources Biologiques de l'Hôpital européen Georges-Pompidou, 20-40 rue Leblanc, 75015 Paris (BRIF number: BB-0033-00063)
- Etablissement Français du sang Hauts de France – Normandie, Site de Bois-Guillaume 609, chemin de la Bretèque, 76235 Bois-Guillaume
- Etablissement Français du sang Nouvelle Aquitaine, site Pellegrin, Place Amélie Raba Léon, CS 21010, 33075 Bordeaux Cedex
- Etablissement Français du sang Hauts de France – Normandie, Site de Loos-Eurasanté, Avenue Pierre Mauroy, Parc Eurasante Epi-de Soil, 59120 Loos
- Etablissement Français du sang Ile de France, Site Avicenne, Hopital Avicenne porte 8, 125 route de Stalingrad, 93009 Bobigny
- Etablissement Français du sang Occitanie, Site de Toulouse, 75 rue de Lisieux, 31300 Toulouse
- Etablissement Français du sang Grand-Est, Site de Colmar, 6 rue du Hohnack, 68025 Colmar Cedex
- Etablissement Français du sang Grand-Est, Site de Metz, 6 rue des Dames de Metz, 57000 Metz
- Etablissement Français du sang PACA-Corse, Site de Marseille, 149, boulevard Baille, 13392 Marseille Cedex 05
